# Time-varying functional connectivity as Wishart processes

**DOI:** 10.1162/imag_a_00184

**Published:** 2024-06-05

**Authors:** Onno P. Kampman, Joe Ziminski, Soroosh Afyouni, Mark van der Wilk, Zoe Kourtzi

**Affiliations:** Department of Psychology, University of Cambridge, Cambridge, United Kingdom; Sainsbury Wellcome Centre for Neural Circuits and Behaviour, University College London, London, United Kingdom; Bayes Centre, University of Edinburgh, Edinburgh, United Kingdom; Optima Partners, London, United Kingdom; Department of Computing, Imperial College London, London, United Kingdom; Department of Computer Science, University of Oxford, Oxford, United Kingdom

**Keywords:** time-varying functional connectivity, functional connectivity, brain connectivity, Wishart processes, functional MRI, methods benchmarking

## Abstract

We investigate the utility of Wishart processes (WPs) for estimating time-varying functional connectivity (TVFC), which is a measure of changes in functional coupling as the correlation between brain region activity in functional magnetic resonance imaging (fMRI). The WP is a stochastic process on covariance matrices that can model dynamic covariances between time series, which makes it a natural fit to this task. Recent advances in scalable approximate inference techniques and the availability of robust open-source libraries have rendered the WP practically viable for fMRI applications. We introduce a comprehensive benchmarking framework to assess WP performance compared with a selection of established TVFC estimation methods. The framework comprises simulations with specified ground-truth covariance structures, a subject phenotype prediction task, a test-retest study, a brain state analysis, an external stimulus prediction task, and a novel data-driven imputation benchmark. The WP performed competitively across all the benchmarks. It outperformed a sliding window (SW) approach with adaptive cross-validated window lengths and a dynamic conditional correlation (DCC)-multivariate generalized autoregressive conditional heteroskedasticity (MGARCH) baseline on the external stimulus prediction task, while being less prone to false positives in the TVFC null models.

## 1 Introduction

The human brain is a dynamic system of interacting regions. Higher-order cognition and complex behavior arise from the spatiotemporal integration, reorganization, and segregation of brain region activity (Bressler & Menon, 2010;Deco et al., 2011;Shine et al., 2016). This intrinsic functional architecture or “connectome” exhibits complex and modular system structures (Deco et al., 2015). Understanding the dynamics of brain region interconnectivity and its changes, for example, during disease, development, or learning, is a critical goal in modern neuroscience.

Functional connectivity (FC) is the study of temporal coactivation across distinct brain regions. This is inferred from the blood-oxygen-level-dependent (BOLD) neural activity signal detected using functional magnetic resonance imaging (fMRI) (Bijsterbosch et al., 2021). These connectivity maps can be used to “fingerprint” subjects, and many inter-individual differences have been identified (Finn et al., 2015;Liégeois et al., 2019). FC is characterized by statistical dependencies (i.e., correlations) between neural activity in brain regions, often assuming a stationary covariance structure (Wang et al., 2014;Zalesky et al., 2012). However, this assumption neglects the complex temporal structure of interregional relationships (Hutchison et al., 2013;Lurie et al., 2020). Therefore, the study of time-varying FC (TVFC) has become increasingly popular (Cohen, 2018;Vidaurre et al., 2021). Instead of estimating a single correlation matrix for an entire scan, correlation is estimated as a function of time during a scan.

Approaches for estimating TVFC include sliding window (SW) methods (Chang & Glover, 2010;Sakoğlu et al., 2010), multiplication of temporal derivatives (Shine et al., 2015), phase coherence models (Glerean et al., 2012), multivariate generalized autoregressive conditional heteroskedasticity (MGARCH) processes (Hakimdavoodi & Amirmazlaghani, 2020;Lindquist et al., 2014), customized Bayesian models (Ebrahimi et al., 2023;Li, Pluta, et al., 2019;Taghia et al., 2017;Warnick et al., 2018), wavelet-based methods (Zhang et al., 2016), and phase synchronization models (Demirtaş et al., 2016;Varela et al., 2001). Another popular approach is to use state-based models, such as the Hidden Markov Model (HMM). Instead of estimating a full covariance structure, each scanning volume is assigned to one of several canonical correlation matrices or “brain states” (Ahrends et al., 2022;Vidaurre et al., 2018). However, all the aforementioned methods exhibit a large degree of variation in their TVFC estimates (Foti & Fox, 2019;Lurie et al., 2020).

This study proposes to estimate TVFC using Wishart processes (WPs). This stochastic process model learns a distribution over (covariance) matrix-valued functions. This is analogous to the Gaussian process (GP) that learns a distribution over scalar-valued functions (Rasmussen & Williams, 2005). Therefore, it is a natural candidate for TVFC modeling. It has recently become possible to adopt this class of models in neuroimaging, thanks to advances in approximate inference routines and easy-to-use computational libraries (Heaukulani & van der Wilk, 2019;Matthews et al., 2017). We discuss the motivation, assumptions, and background of this model; how to construct it; how to employ Bayesian inference to fit its parameters; and its potential advantages and limitations.

Evaluating TVFC estimation methods remains difficult because the ground-truth covariance structure in real data is unobserved. Therefore, estimation methods coexist, despite the large degree of variation in their estimates. To evaluate the application of WPs to covariance estimation between fMRI time series, we designed a comprehensive collection of benchmarks that serve as proxies for the direct observation of true TVFC. Each benchmark comprises a competitive prediction task in which the WP is compared with other TVFC estimation methods (Leenings et al., 2022;Yarkoni & Westfall, 2017). We study established prediction tasks including simulation benchmarks, where time series are generated using prespecified underlying covariance structures (Hindriks et al., 2016;Thompson et al., 2018) and where the prediction task is to “reconstruct” this ground-truth covariance structure from the simulated observations, resting-state fMRI (rs-fMRI) benchmarks, where we study the predictive power of estimated TVFC of subject measures and their test-retest robustness, and task-based fMRI (tb-fMRI) benchmarks, where the goal is to predict the presence of an external stimulus (Gonzalez-Castillo & Bandettini, 2018;Sahib et al., 2018). Additionally, we introduce a data-driven imputation benchmark, in which the prediction task is to infer TVFC at a left-out test set of observations using observations from the remaining training dataset.

InSection 2, we describe the existing TVFC estimation methods that serve as baselines for comparison with the WP. These include the static FC (sFC) estimate, an improved version of the SW approach using cross-validated window lengths (SW-CV), and the dynamic conditional correlation (DCC) MGARCH model. Next, we discuss how we constructed the WP and inferred its model parameters. We then discuss the benchmarks that were studied. After presenting the results inSection 3, we summarize the insights into WP performance across these benchmarks inSection 4. We discuss limitations and provide promising avenues for future work to address these.

## 2 Methods

In this section, we describe methods for time-varying covariance estimation, how to extract features from estimated covariance structures, and how to compare estimation method performance through benchmarking.

### 2.1 Baseline TVFC estimation methods

#### 2.1.1 Static functional connectivity (sFC)

Static connectivity estimates assume that the covariance between the random variables of interest does not change across any arbitrary scanning session length. The multivariate BOLD observations at time step (i.e., scanning volume)1≤n≤Nfor activity time series fromDregions (or*nodes*) are denoted as${\text{y}}_{n}\in {\mathbb{R}}^{D}$. As sample means are assumed to be zero, the (unbiased) covariance matrix estimator is

$$\hat{\Sigma }=\frac{1}{N-1}\;\sum_{n=1}^{N}{\text{y}}_{n}\;{\text{y}}_{n}^{T}.$$ (2. 1)

The respective (Pearson) correlation matrix is obtained by dividing each covariance matrix element by the product of the standard deviations of the respective variables.

#### 2.1.2 Sliding window (SW) functional connectivity

SW methods slide a time window of a certain lengthωand shape (typically rectangular or Gaussian) across observations. The covariance is estimated within this window for each step (Shakil et al., 2016). The window length is typically between 30 and 60 seconds (Shirer et al., 2012). In this study, we implemented a rectangular window with a step size of a single volume. Time series were high-pass filtered (5^th^-order Butterworth) to remove the frequency components below1/ωprior to TVFC estimation (Leonardi & Van De Ville, 2015). Zeros were padded to the start and end of the time series to allow for estimation around those locations.

**Fig. 1.**
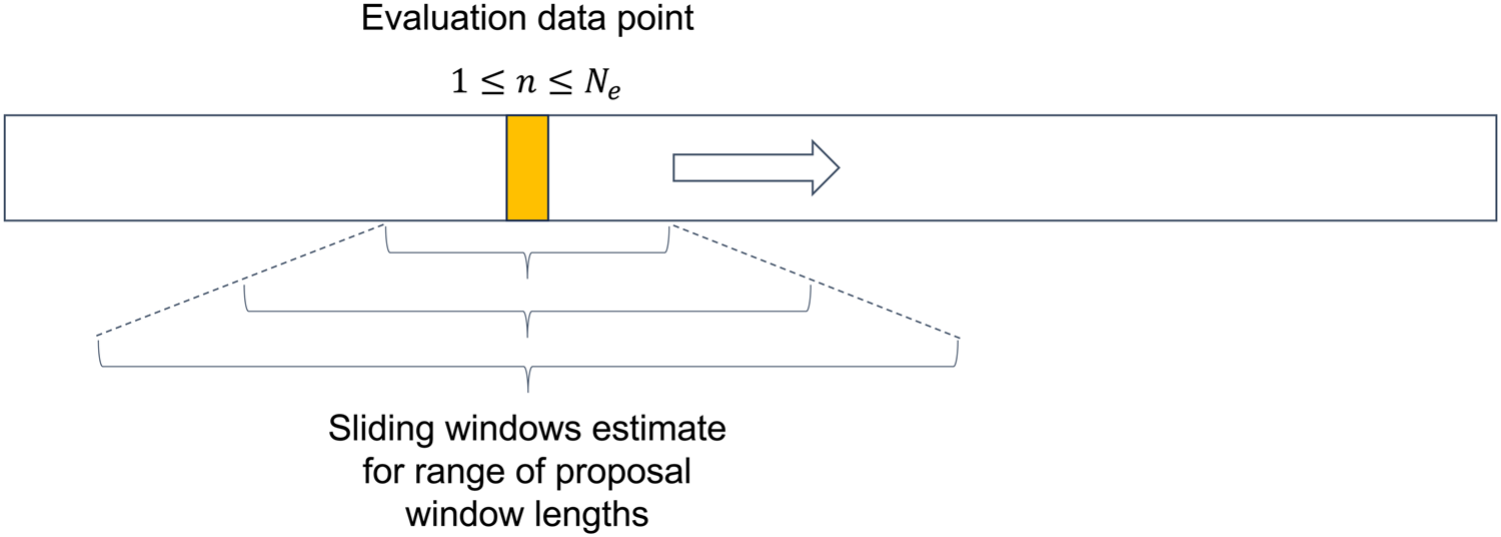
Schematic of determining the optimal window length for sliding window (SW) TVFC estimation using cross-validation. A range of proposed window lengths was used to estimate TVFC around a range of evaluation data points. The optimal window length is determined as the one whose estimates yield the highest likelihood under a zero-mean Gaussian distribution, averaged over all evaluation data points.

Window lengths affect the estimated TVFC. Small windows can detect fast-changing aspects of the data but often return spurious covariance, whereas large windows are more robust to outliers but are unable to detect faster dynamics. Real datasets contain unknown temporal structures, and each scan may require a different window length. Strategies exist to determine the window length a priori or to circumvent the choice of window length (Nielsen et al., 2017;Vergara et al., 2019;Yaesoubi et al., 2018), such as choosing a window length to avoid1/ωexceeding the Nyquist frequency (Zalesky et al., 2014). However, a consensus strategy for selecting this hyperparameter is lacking. In this study, we posited that good methods for inferring TVFC will also be able to predict TVFC at unseen time points. We implemented a data-driven approach for optimal a priori window length selection using cross-validation (Fig. 1). The evaluation data points were obtained one at a time from the time series as a validation set. The average log likelihood of these observations under a zero-mean multivariate Gaussian distribution was then computed as

$$ℒ=\frac{1}{N_{e}}\sum_{n}^{N_{e}}\left[-\frac{D}{2}\log2\text{π}-\frac{1}{2}\log|{\hat{\Sigma }}_{n}|-\frac{1}{2}{\text{y}}_{n}^{\text{T}}{\hat{\Sigma }}_{n}^{-1}{\text{y}}_{n}\right],$$ (2. 2)

where$1\leq n\leq N_{e}$is an evaluation data point,$N_{e}$denotes the number of evaluation points (equal to the total number of time stepsNminus the maximum proposal window length$\omega_{max}$), and${\hat{\Sigma }}_{n}$(a function ofω) is the covariance matrix estimate for all observations in the surrounding window of lengthω(*excluding*the evaluation data point). This procedure was repeated for a range of proposed window lengths. The optimal window length$\hat{\omega }$maximizes the log likelihood:

$$\hat{\omega }=\underset{\omega_{\min}\;\leq \;\;\omega \;\;\leq \;\;\omega_{\max}}{\text{arg max}}\frac{1}{N_{e}}\;\sum_{n}^{N_{e}}\left[-\frac{D}{2}\log2\text{π}-\frac{1}{2}\log|{\hat{\Sigma }}_{n}|\;-\frac{1}{2}{\text{y}}_{n}^{\text{T}}{\hat{\Sigma }}_{n}^{-1}{\text{y}}_{n}\right].$$ (2. 3)

The minimum and maximum window lengths to explore ($\omega_{min}$and$\omega_{max}$) were set to 20 (Leonardi & Van De Ville, 2015) and 180 seconds, respectively. For example, assuming a repetition time (TR) of 2 seconds, a window would include 10–90 data points. We empirically verified that this approach consistently outperformed hard-coded window lengths (seeSupplementary Materials Sections S.1 and S.7).

#### 2.1.3 MGARCH: Dynamic conditional correlation (DCC)

The MGARCH framework, which is popular in both econometrics and neuroimaging, describes a family of models that considers a zero-mean vector stochastic process${\text{y}}_{n}\in {\mathbb{R}}^{D}$with a time-varying covariance structure:

$${\text{y}}_{n}=\sum_{n}^{1/2}\eta_{n},$$ (2. 4)

where$\eta_{n}$is an i.i.d. white noise vector process. The variances of the individual time series are assumed to follow a vector autoregression process; therefore, the dynamic covariance process$\sum_{n}$is conditioned on all observations up until timen−1. In this study, we considered the DCC-GARCH model (Engle, 2002). Specifically, we implemented a first-order DCC(1,1) model as commonly used in FC studies (Choe et al., 2017;Lindquist et al., 2014). Parameter inference starts by fitting a univariate GARCH model (Bollerslev, 1986;Engle, 1982) to each time series individually, for which we used a first-order GARCH(1,1) process, meaning each state only considers both the GARCH and ARCH terms from the preceding state.

When estimating the covariance structure between a set ofD>2time series, we could either model time series jointly or loop over all pairs of time series, trainingD(D−1)/2models and thus learning more parameters. Whether different parameters should be learned for each node pair (or*edge*) depends on whether we expect significantly different dynamic characteristics between edges. In FC studies, running DCC in a pairwise manner is common (Choe et al., 2017). However, DCC models were designed to model all dimensions jointly. The optimal implementation remains unclear. Therefore, we included both: throughout this paper, “DCC-J” indicates a (multivariate) jointly trained model and “DCC-BL” refers to bivariate loop (pairwise) training.

### 2.2 The Wishart process

#### 2.2.1 Model construction

We constructed the WP as a single statistical model that describes the time-varying covariance structures between time series. To start, the Wishart distribution defines a probability density function over positive definite matrices∑:

$$f\left(\sum \text{|}\text{V},\nu \right)=\frac{1}{Z}{\left|\sum \right|}^{\left(\nu -D-1\right)/2}\exp\left(-\frac{1}{2}tr\left({\text{V}}^{-1}\sum \right)\right),$$ (2. 5)

whereVis a*D×D*positive definite scale matrix,ν≥Dis the number of degrees of freedom, and with normalization constant$Z=2^{\nu D/2}{\left|\text{V}\right|}^{\nu /2}\Gamma_{D}\left(\nu /2\right)$, where$\Gamma_{D}\left(\cdot \right)$is the multivariate gamma function. This distribution is a multivariate extension of the chi-squared distribution and is used extensively in the estimation of covariance matrices in multivariate statistics. Crucially, we can construct a Wishart-distributed random (matrix-valued) variable from a collection of i.i.d. zero-mean multivariate Gaussian random variables. The sum of the outer products of such variables is Wishart distributed:

$$\sum =\sum_{i\;=\;1}^{\nu }{\text{u}}_{i}{\text{u}}_{i}^{T}∼W_{D}\left(\text{V},\nu \right),$$ (2. 6)

where${\text{u}}_{i}$are i.i.d.N(0,V)distributed,D-dimensional random variables, and$W_{D}\left(\text{V},\nu \right)$denotes a Wishart distribution with a scale matrixVandνdegrees of freedom.

The WP is a continuous time stochastic process on symmetric positive definite matrices, which makes it suitable for modeling covariance matrices (Bru, 1991;Wilson & Ghahramani, 2011). We let$Y:\;=\left({\text{y}}_{n},1\leq n\leq N\right)$denote a sequence of BOLD measurements in${\mathbb{R}}^{D}$, whereNis the number of scan volumes andDis the number of time series. Index locations are denoted as$X:\;=\left(x_{n},1\leq n\leq N\right)$inℝas the times at which the measurement${\text{y}}_{n}$is observed, considered in a fixed interval of [0, 1] during training and prediction. These index locations were univariate in our case, but could be multivariate when other covariates, such as head motion, would be included. We chose the conditional likelihood of observations to be a multivariate Gaussian:

$${\text{y}}_{n}|\mu_{n},\sum_{n}∼N\left(\mu_{n},\sum_{n}\right),$$ (2. 7)

where$\sum_{n}$is aD×Dcovariance matrix and$\mu_{n}=\text{0}$in our context. The random and unobserved process$\sum_{1},\sum_{2},\ldots ,$$\sum_{N}$constitutes TVFC. Analogous to constructing a Wishart-distributed random variable from a collection of Gaussian random variables, we constructed the Wishart*process*from i.i.d. collections of Gaussian*processes*. Let

$$f_{d,k}∼GP\left(\text{0},K\left(\cdot ,\cdot ;\theta \right)\right),$$ (2. 8)

for1≤d≤Dand1≤k≤ν, whereK(⋅,⋅;θ)denotes a kernel function. Let$F_{n,d,k}:\;=f_{d,k}\left(X_{n}\right)$denote the evaluation of the GP at$X_{n}$. We wrote${\text{F}}_{n}$for the aggregateD×νmatrix${\left(F_{n,d,k}\right)}_{1\leq d\leq D,1\leq k\leq \nu }$, which has entries$F_{n,d,k}$indexed bydandk. Analogues toEq. (2.6), we construct

$$\sum_{n}=\text{A}{\text{F}}_{n}{\text{F}}_{n}^{T}{\text{A}}^{T}$$ (2. 9)

as a Wishart-distributed random matrix at time1≤n≤N. This construction allows us to query covariance matrices continuously throughout an fMRI scan, because the underlying GPs can be queried at any time. It also handles missing data naturally because data are not expected in a grid-like pattern. Matrix$\text{A}\in {\mathbb{R}}^{D\times D}$is restricted such that the scale matrix$\text{A}{\text{A}}^{T}$is positive definite, and it is trained as part of the inference routine. This scale matrix provides the expected value of∑for alln. The log likelihood fromEq. (2.7)with this construction of$\sum_{n}$plugged in is

$$\begin{array}{l} \log p\left({\text{y}}_{n}\text{|}\;\text{A},{\text{F}}_{n}\right)=-\frac{D}{2}\log\left(2\text{π}\right)-\frac{1}{2}\log\left|\text{A}{\text{F}}_{n}{\text{F}}_{n}^{T}{\text{A}}^{T}\right|\\ \;\;\;\;\;\;\;\;\;\;\;\;\;\;\;\;\;\;\;\;\;\;\;\;\;\;\;\;\;\;\;\;\;\;\;\;\;\;\;\;\;\;\;\;\;-\frac{1}{2}{\text{y}}_{n}^{T}{\left(\text{A}{\text{F}}_{n}{\text{F}}_{n}^{T}{\text{A}}^{T}\right)}^{-1}{\text{y}}_{n}. \end{array}$$ (2. 10)

Heaukulani and van der Wilk (2019)suggested adding white noise for robust inference, which we validated empirically. The covariance matrix construction is updated to

$$\sum_{n}=\text{A}{\text{F}}_{n}{\text{F}}_{n}^{T}{\text{A}}^{T}\;+\Lambda ,$$ (2. 11)

with additive noise matrixΛ: a diagonalD×Dmatrix with positive entries, whose values are trained with the rest of the parameters (off-diagonal values remain zero).

#### 2.2.2 Bayesian inference

After defining our model, we needed a procedure to fit it. Doing so requires computingp(Y)=∫p(Y | F)p(F)dFin the posterior$p\left(F\text{|}Y\right)=\frac{p\left(Y,F\right)}{p\left(Y\right)}$, which is intractable. Therefore, we used variational inference, a technique that approximates the probability density through optimization (Blei et al., 2017). This contrasts with Markov Chain Monte Carlo methods, which are often employed in such situations. Variational inference posits a family of distributionsq(F)over the latent variables and then finds the member of that family that is close in terms of Kullback-Leibler (KL) divergence to the target distribution (the true posterior). Importantly, model parameters and covariance structure aspects are learned directly from the data instead of having fixed hyperparameters representative of inductive biases on behalf of the practitioner.

To make our WP models more computationally viable, we used*sparse*underlying variational GPs as WP building blocks. The sparse variant introduces the concept of inducing points: learned auxiliary data points (Bauer et al., 2016;Titsias, 2009). The number of inducing points$Z:\;=\left(z_{m},1\leq m\leq M\right)$is smaller thanN. We write$U_{m,d,k}:\;=f_{d,k}\left(Z_{m}\right)$for sparse GP evaluations. We chose a fully factorized Gaussian prior over$U_{d,k}$. We collectively denote$U_{d,k}:\;=\left(U_{m,d,k},m\leq M\right)$and chose its variational approximation as

$$q(U_{d,k})∼N(U_{d,k};\mu_{d,k},{\text{S}}_{d,k}),$$ (2. 12)

for variational parameters$\mu_{d,k}\in {\mathbb{R}}^{M}$and${\text{S}}_{d,k}\in {\mathbb{R}}^{M\times M}$a real, symmetric, positive definite matrix. For inference, we iteratively maximized the evidence lower bound (ELBO)

$$\begin{array}{l} ELBO=\;\;\sum_{n\;=\;1}^{N}E_{q\left({\text{F}}_{n}\right)}\left[\log p\left({\text{y}}_{n}\text{|}\;{\text{F}}_{n}\right)\right]\\ \;\;\;\;\;\;\;\;\;\;\;\;\;\;\;\;\;\;-\sum_{d=1}^{D}\sum_{k=1}^{\nu }KL\left(q\left(U_{d,k}\right)||p\left(U_{d,k}\right)\right) \end{array}$$ (2. 13)

as our objective function using gradient descent, where (approximate) gradients were computed based on samples (Monte Carlo estimates) of our objective function. Intuitively, the first ELBO term is a likelihood (data fit) term and the second is a regularizing term.

#### 2.2.3 Implementation

The properties of the underlying GPs are determined by a kernel function, for which we used a (stationary, isotropic) Matérn 5/2 kernel

$$k\left(\text{x},{\text{x}}^{\prime }\right)=\sigma^{2}\left(1+\sqrt{5}r+\frac{5}{3}r^{2}\right)\exp\left(-\sqrt{5}r\right),$$ (2. 14)

with$r=\frac{\|\text{x}-{\text{x}}^{\prime }\|}{l}$, and whereland$\sigma^{2}$are the kernel length scales and variance parameters, respectively. This length scales parameter is easily interpretable and defines how quickly the GP (and, therefore, TVFC as a WP) can change over time by how much weight the model places on observations farther from the evaluation index location. Therefore, we hypothesized that this parameter may be analogous to the window length in SW (seeSupplementary Materials Section S.7).

Parameters were updated using gradient descent with the Adam optimizer (Kingma & Ba, 2015). The estimated parameters include the variational parameters, kernel hyperparameters, the lower Cholesky decompositionAof the scale matrix, and the diagonal values of the additive noise matrixΛ. We setν=DandM=200. All figures include the 95% confidence interval of the mean estimate plus/minus two standard deviations, based on 3,000 samples from the posterior. Although this model is complex in its description, and thus introduces a complexity penalty, its*implementation*is lean and robust, relying heavily on open-source libraries. Further, while there is no theoretical limit to the number of time series modeled, the computational demand for training this model on high-dimensional data (over 100 time series) is high, taking days or even weeks for a single scan. In practice, this means researchers working with fMRI may need access to sizeable compute clusters (e.g., parallelizing training bivariate models for each edge) or GPUs. Alternatively, they may run a dimensionality reduction algorithm before fitting a WP to their data.

### 2.3 Benchmarking TVFC estimation methods

#### 2.3.1 Simulation benchmarks

**Fig. 2.**
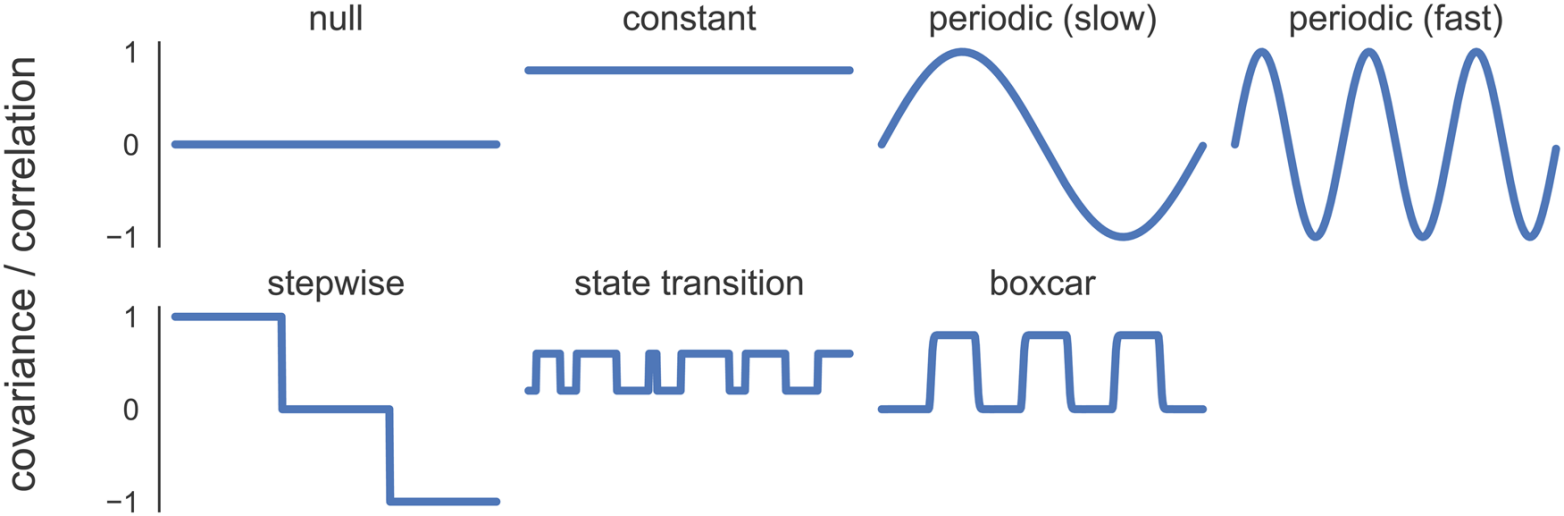
Synthetic covariance structures as a function of time for simulation benchmarks. This set was designed to capture a wide range of edge cases and potentially realistic underlying covariance structures. Synthetic activity time series were generated from these covariance structures.

We considered simulated data from seven underlying deterministic synthetic covariance structures (Fig. 2):**null**covariance (time series are uncorrelated), static**constant**covariance of$\sigma_{ij}=0.8$, two**periodic**covariance structures (a**slow**and a**fast**oscillating sine wave defined by one and three periods, respectively) that model transient changes in coupling,**stepwise**covariance that models two large change points in covariance, a covariance structure that mimics a series of**state transitions**(Thompson et al., 2018), and a covariance structure inspired by a**boxcar**external task design. For each of these, we sampled the observations${\text{y}}_{n}\in {\mathbb{R}}^{D}$at time steps1≤n≤Nfrom a zero-mean Gaussian distribution:

$${\text{y}}_{n}∼N\left(0,\sum_{n}\right),$$ (2. 15)

for the respective covariance matrices. We tested on pairwise (i.e., bivariate) and trivariate datasets. The covariance structure for generating bivariate data was

$$\sum_{n}=\left[\begin{array}{cc} 1 & \sigma \left(n\right)\\ \sigma \left(n\right) & 1 \end{array}\right],$$ (2. 16)

where the covariance termσ(n)varied with time according to the synthetic covariance structures (Fig. 2). For the trivariate data, both a*dense*, fully covarying covariance structure

$$\sum_{n}=\left[\begin{array}{ccc} 1 & \sigma \left(n\right) & \sigma \left(n\right)\\ \sigma \left(n\right) & 1 & \sigma \left(n\right)\\ \sigma \left(n\right) & \sigma \left(n\right) & 1 \end{array}\right],$$ (2. 17)

and a*sparse*version where only the first two time series are correlated

$$\sum_{n}=\left[\begin{array}{ccc} 1 & \sigma \left(n\right) & 0\\ \sigma \left(n\right) & 1 & 0\\ 0 & 0 & 1 \end{array}\right],$$ (2. 18)

were implemented. The range ofσ(n)was updated to [−0.5, 1] to maintain the covariance matrices as positive semidefinite in the dense case. The time series were individually normalized to have a mean of zero and a unit standard deviation. Model performance was averaged acrossT=200trials. We report the results ofN=400scanning volumes. The results for higher dimensions can be found inSupplementary Materials Section S.2.

Noise with a signal-to-noise ratio (SNR) of 2 was added to these time series. The noisy${\text{y}}_{n}^{*}$was constructed as a linear mix of noiseless signal${\text{y}}_{n}$(Eq. 2.15) and (independent) noise time series$\epsilon_{n}$:

$${\text{y}}_{n}^{*}=\alpha {\text{y}}_{n}+\left(1-\alpha \right)\epsilon_{n},$$ (2. 19)

where0<α<1and the SNR is equal to$\frac{\alpha }{1-\alpha }$. Noisy time series more realistically mimic fMRI data, especially when the noise contains properties as found in fMRI data (Deco et al., 2009;Welvaert & Rosseel, 2013). Therefore, we used data from the Human Connectome Project (HCP) (Elam et al., 2021;Smith, Beckmann, et al., 2013) as$\epsilon_{n}$; a source of noise. A detailed description of HCP data can be found inAppendix A.2. The time series obtained from the HCP were representative of an independent component analysis (ICA) component of whole-brain activity. For eachD-dimensional set of simulated time series (i.e., individually for each covariance structure and trial), we selected the time series fromDrandomly selected ICA components from a randomly selected scan of*different*HCP subjects. As they were obtained from different subjects, these noise time series could be assumed to be uncorrelated. This ensured that no additional and adversarial (i.e., other than the predetermined “ground truth”) covariance structure was introduced. These noise time series were preferred over other types of noise, such as white noise, as they preserve realistic fMRI data characteristics such as spatial and temporal autocorrelation (Arbabshirani et al., 2014;Honari et al., 2019;Keilholz et al., 2017). The addition of noise affects the ground-truth variances and covariances, which were updated accordingly (Appendix A.1). The root mean square error (RMSE) between the estimated and ground-truth correlations served as the performance metric (Lindquist et al., 2014;Wilson & Ghahramani, 2011). For the trivariate cases, we computed the RMSE across all elements of the full correlation matrices.

#### 2.3.2 Resting-state fMRI benchmarks

We studied the capability of predicting subject measures, test–retest robustness, and a brain state analysis using HCP rs-fMRI data. Each activity time series in this dataset represented an ICA component of whole-brain activity. For interpretable features, we computed edgewise (i.e., independently for each connection between nodes) summary measures of TVFC estimates across time: the mean (analogous to sFC estimates; considered the connection “strength”)

$$\mu_{i,j}=\frac{1}{N}\sum_{n=1}^{N}\sum_{n,\;i,j},$$ (2. 20)

the variance (Choe et al., 2017;Hutchison et al., 2013), sometimes interpreted as connectivity “stability” or “flexibility” (Allen et al., 2014)

$$\sigma_{i,j}^{2}=\frac{1}{N}\sum_{n=1}^{N}{\left(\sum_{n,i,j}-\mu_{i,j}\right)}^{2},$$ (2. 21)

and we proposed an additional summary measure, the TVFC*rate-of-change*, defined as the mean absolute relative difference between subsequent time steps

$$r_{i,j}=\frac{1}{N-1}\sum_{n=2}^{N}\left|\frac{\sum_{n,\;i,j}}{\sum_{n-1,\;i,j}}-1\;\right|,$$ (2. 22)

for the edge between nodes1≤i≤Dand1≤j≤D. The latter summary measure captured how smoothly the estimated TVFC changed over time.

The estimated TVFC was related to subject measures using a morphometricity analysis (Sabuncu et al., 2016), but instead of anatomical variation between subjects, we studied variation in TVFC. We posited the linear mixed effects model

y=Xβ+a+ϵ, (2. 23)

whereyis a column vector of length equal to the number of subjects studied containing some quantitative subject measure,Xis a design matrix of nuisance variables (covariates) weighted by β,$\text{a}\sim N(\text{0},\sigma_{a}^{2}{\text{K}}_{a})$is a random effects vector, and$\epsilon \sim N(\text{0},\sigma_{e}^{2})$is a noise vector. Entries in the symmetric matrix***${\text{K}}_{a}$***encoded how globally “similar” the TVFC estimates between the respective subjects were. This similarity was defined using the three summary measures (including two dynamic measures) of estimated whole-brain TVFC, taking the lower triangular values as a vector of sizeD(D−1)/2. The similarity “distance” between subject vectors was defined by (nonlinear) Gaussian kernel$k(x,x^{\prime })=\exp(-{\left(x-x^{\prime }\right)}^{2})$. Morphometricity was computed as the proportion of phenotypic variation that could be explained by intersubject variation:

$$m^{2}=\frac{\sigma_{a}^{2}}{\sigma_{a}^{2}+\sigma_{e}^{2}}=\frac{\sigma_{a}^{2}}{\sigma_{y}^{2}},$$ (2. 24)

with$\sigma_{y}^{2}$the phenotypic variance. Parameters$\sigma_{a}^{2}$and$\sigma_{e}^{2}$were estimated using the restricted maximum likelihood method (Harville, 1977). The analysis was repeated for 15 subject measures, including age, gender, and cognitive task scores (Kong et al., 2019;Li, Kong, et al., 2019). Age and gender were included as nuisance variables (when they were not predicted).

Further, we compared the robustness of the TVFC estimates across scans from the same subject (Abrol et al., 2016,^2017^;Choe et al., 2017;Elliott et al., 2020;Noble et al., 2019). The test–retest reliability of TVFC summary measures was computed as interclass correlation coefficients (ICCs), individually for each edge (Chen et al., 2018;Shrout & Fleiss, 1979):

$$ICC=\frac{\sigma_{X}^{2}}{\sigma_{X}^{2}+\sigma_{U}^{2}},$$ (2. 25)

with$\sigma_{X}^{2}$the between-subject and$\sigma_{U}^{2}$the within-subject variance. We computed image intraclass correlation coefficients (I2C2) as a whole-brain reliability assessment (Shou et al., 2013):

$$I2C2=\frac{tr\left({\text{K}}_{X}\right)}{tr\left({\text{K}}_{X}+{\text{K}}_{U}\right)},$$ (2. 26)

with${\text{K}}_{X}\in {\mathbb{R}}^{D(D-1)/2\times D(D-1)/2}$the within-subjects covariance (of “true” FC) and${\text{K}}_{U}\in {\mathbb{R}}^{D\left(D-1\right)/2\times D\left(D-1\right)/2}$the covariance of the measurement error vector (the difference between measurement and “true” FC). These matrices were computed using method of moments estimators (Carroll et al., 2006;Shou et al., 2013). Values for both ICC and I2C2 lie between 0 and 1, where 0 represents complete retest independence and 1 represents perfect measurement reliability.

Finally, TVFC estimates are commonly characterized as switching between recurring FC correlation matrices or “brain states” (Keilholz et al., 2017;Kringelbach & Deco, 2020). At any point in time, a subject’s functional organization is defined by one ofkbrain states, between which a subject traverses during the scanning session. In this study, for each session separately, all estimated correlation matrices for allNtime steps and all subjects were concatenated, and brain states were extracted using thek-means algorithm (Lloyd, 1982). We extractedk=3brain states, followingChoe et al. (2017). We also computed the number of brain state change points (i.e., switches between states within a scan). The brain state analysis was not considered as a benchmark for method selection, because it was unclear how to determine the superior TVFC estimation method from this analysis.

#### 2.3.3 Task-based fMRI benchmarks

We assessed which method’s TVFC estimates could best predict an external task stimulus of brain activity, using an elegantly simple visual task experiment from the Rockland dataset (Nooner et al., 2012). More details on this dataset and the task paradigm can be found inAppendix A.3. Predicting the presence or absence of the visual stimulus was framed using a generalized linear model (GLM)

$${\text{y}}_{\text{g}}=\text{X}\beta_{\text{g}}+{\text{e}}_{\text{g}},$$ (2. 27)

where${\text{y}}_{g}\in {\mathbb{R}}^{N}$is the estimated TVFC for a given method, which was run independently for all1≤g≤Gedges of interest (i.e., the connectivity between the primary visual cortex (V1) and five other ROIs; V2, V3, V4, mPFC, and M1),$\text{X}\in {\mathbb{R}}^{R\text{x}N}$is the design matrix withR=3regressors (a single experimental regressor and two nuisance regressors) weighted by$\beta \in {\mathbb{R}}^{R}$, and${\text{e}}_{\text{g}}\in {\mathbb{R}}^{N}$are the error terms. The experimental regressor was the boxcar (block design) model of the visual stimulus convolved with a hemodynamic response function (HRF) (Glover, 1999). The two nuisance regressors were first-order polynomials, where the polynomial (drift) order was determined as the TR multiplied by$\frac{N}{150}$(Worsley et al., 2002). We inferredβfrom the observations, using ordinary least squares (OLS) to minimize the error terms (Worsley & Friston, 1995). The magnitudes ofβparameters were then used to determine which TVFC estimates best captured the presence of the visual stimulus.

#### 2.3.4 The imputation benchmark

**Fig. 3.**
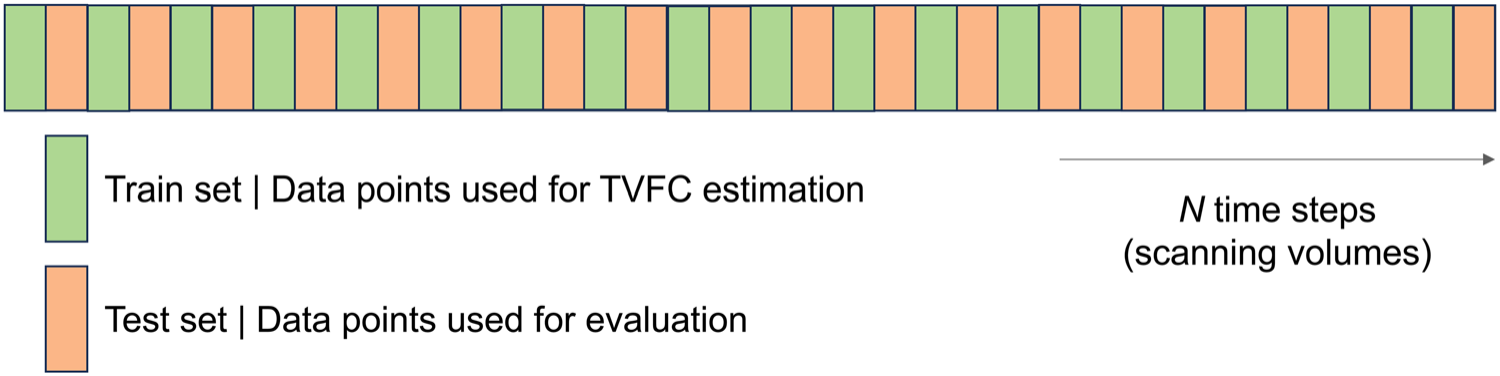
Schematic of the imputation benchmark. The time series were split into equally sized training and test sets. Training set data points were used to infer TVFC at the test set locations. Benchmark performance was the log likelihood of observing the test set under a zero-mean Gaussian distribution defined by the inferred TVFC, averaged over all test set data points.

Our proposed imputation benchmark is a data-driven benchmark for comparing TVFC estimation methods that only uses the observed time series. Again, we posited that good TVFC estimation methods can predict TVFC at unseen time points. Training and test sets were defined as data points taken from the time series under a leave-every-other-out (LEOO) split, resulting in equally sized training and test sets (Fig. 3). Each candidate method was tasked with estimating the covariance structure at the test locations using only observations from the training set. Except for the WP, where interpolation follows naturally, the test location covariance matrix estimates were elementwise and linearly interpolated between the estimates at the enclosing training locations. The performance metric was the mean log likelihood of observing the test set observations under a zero-mean Gaussian distribution defined by the estimated covariance matrices. We applied this benchmark to all datasets enclosed in this study.

## 3 Results

### 3.1 Simulation benchmarks

First, we investigated the ability of the TVFC estimation methods to recover known covariance structures from simulated multivariate time series. Simulation benchmarks address a significant obstacle in assessing TVFC in fMRI data: the lack of ground-truth knowledge of the covariance structure. We tested against a battery of synthetic covariance structures that could plausibly be encountered in an fMRI scan (Fig. 2):**null**covariance,**constant**static covariance,**periodic (slow and fast)**covariance structures of an oscillating sine wave (slow, low frequency—one period; fast, high frequency—three periods),**stepwise**covariance that models two large change points, a covariance structure that mimics a series of**state transitions**(Thompson et al., 2018), and a covariance structure inspired by a blocked-design**boxcar**tb-fMRI study that mimics the presence and absence of an external stimulus, as modeled by convolving a square wave stimulus time series with an HRF.

To investigate the qualitative differences between the estimation methods, the bivariate TVFC estimates for a single trial for all covariance structures are shown inFigure 4. We quantified the performance by computing the RMSE between the estimated and ground-truth correlation terms (i.e., the off-diagonal term;Figure 5, top row). Differences between the methods were statistically tested using two-tailed*t*-tests with Bonferroni correction. Of note is the spurious covariance detected by SW-CV in the static**null**and**constant**cases (Hindriks et al., 2016;Lindquist et al., 2014). The WP and DCC methods performed comparably to sFC, with the SW-CV performing worse than the WP (t=−22.00,$p=9.41\times 10^{-71}$, andt=−9.67,$p=5.3\times 10^{-20}$, for the null and constant covariance structures, respectively). For the two**periodic**(**slow**and**fast**) covariance structures, the smooth nature of the WP was particularly suitable, in contrast to the DCC estimates, where large, spurious jumps were observed (t=−25.87,$p=2.9\times 10^{-87}$, andt=−34.76,$p=1.2\times 10^{-122}$, for the slow and fast periodic covariance structures, respectively). However, the WP estimates were too smooth to capture the sudden changes in the**stepwise**covariance structure. The SW-CV estimates suffered from a similar problem, where static parts require longer windows, but the change points require shorter windows. While DCC could model the change points, it returned spurious covariance estimates in the static parts of the time series. All methods failed to reconstruct the**state transition**covariance structure, as evidenced by the fact that none of the methods were able to significantly outperform the sFC estimate (Fig. 5, top row). This was interpreted as none of the methods was sensitive enough to detect subtle yet sudden changes in the covariance structure. All methods could model the**boxcar**covariance structure; however, performance was reduced when noise was added. The SW-CV performed better than the WP on this dynamic covariance structure (t=13.77,$p=1.4\times 10^{-35}$).

**Fig. 4.**
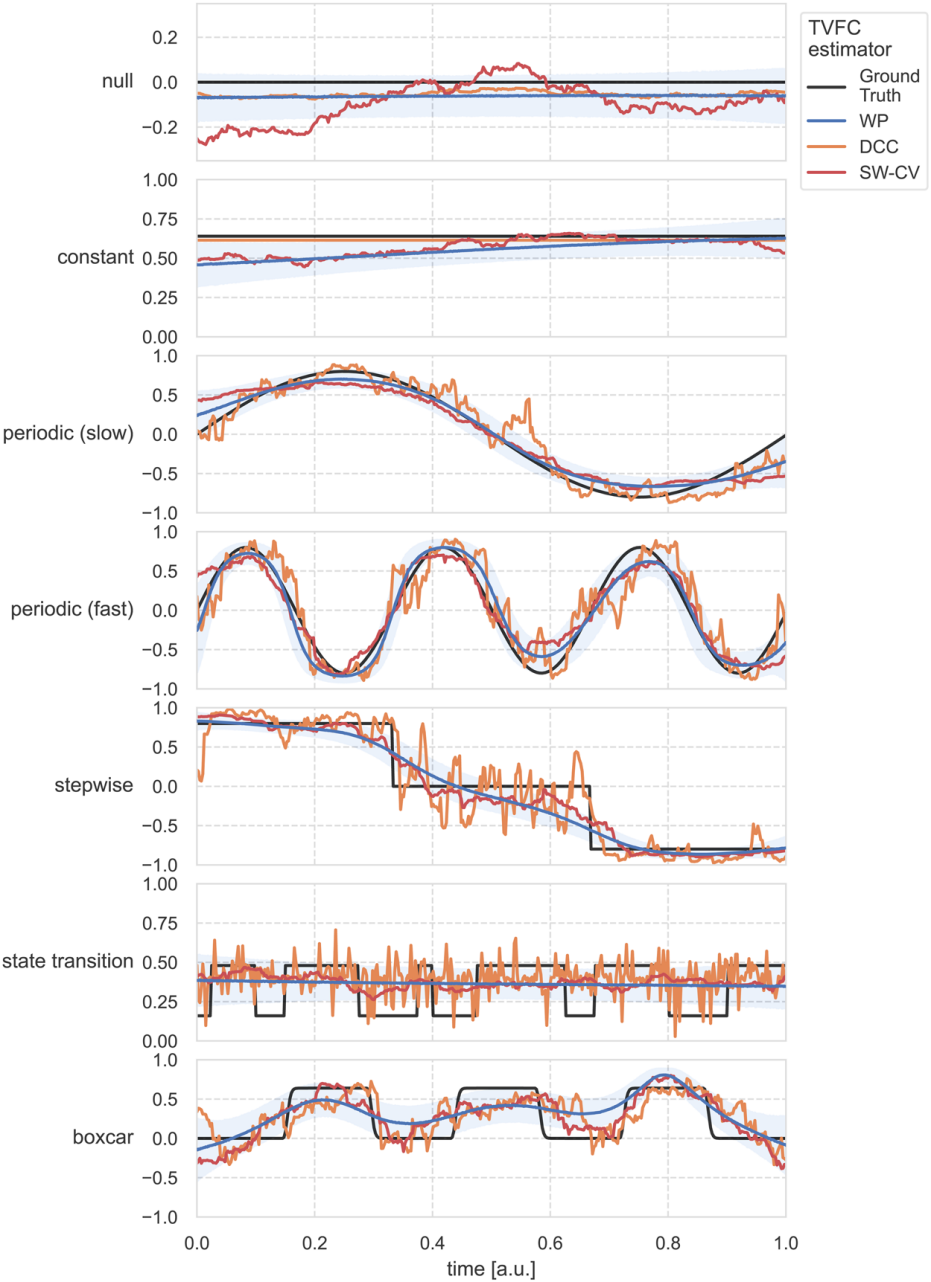
Simulation benchmarks single trial bivariate TVFC estimates from the methods considered forN=400time steps. The estimation methods have distinct failure modes. All methods struggle with the change points in the covariance structures. WP, Wishart process; DCC, dynamic conditional correlation; SW-CV, cross-validated sliding windows.

**Fig. 5.**
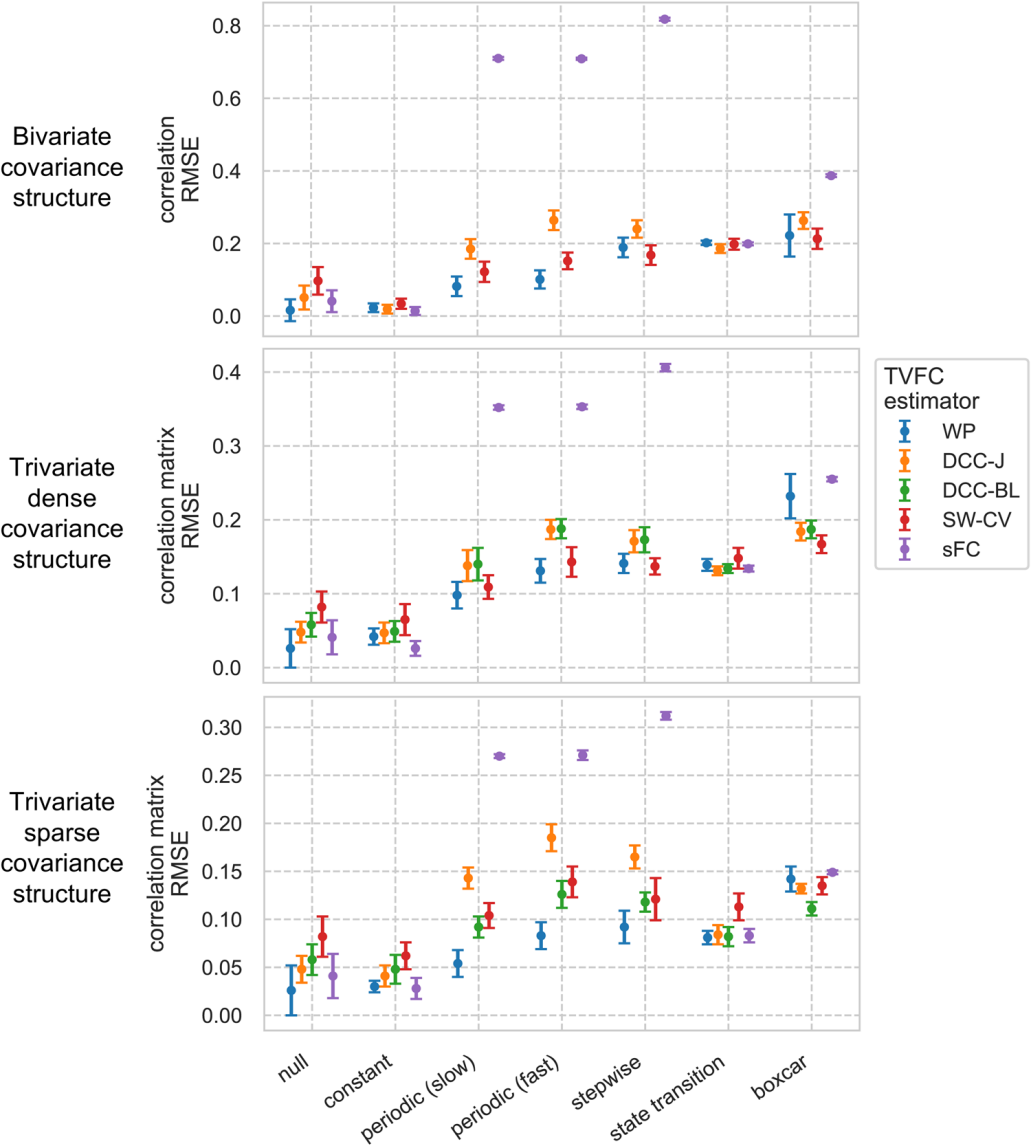
Simulation benchmarks quantified results for bivariate (top row), dense trivariate (middle row), and sparse trivariate (bottom row) scenarios. Performance was quantified as the root mean squared error (RMSE) between the TVFC estimates and ground truth for all prespecified covariance structures. The performance of sFC estimates is included for reference. Shown are the results for the simulated data with added rs-fMRI noise (SNR of 2) for*N=400*time steps. Means and standard deviations are shown acrossT=200trials. WP, Wishart process; DCC, dynamic conditional correlation, trained in joint (“-J”) and bivariate loop (“-BL”) fashion; SW-CV, cross-validated sliding windows; sFC, static functional connectivity.

We undertook further benchmarking in the trivariate case (Fig. 5, middle and bottom row). Both a dense version, where all time series edges share the same correlation structure, and a sparse case, where the third time series is uncorrelated with the first two time series, were implemented. Competitive performance, measured as the RMSE between all elements of the estimated and ground-truth correlation matrices, was observed for WP estimates. Similar to the bivariate case, SW-CV estimates return a time-varying structure when the ground-truth covariance is static (e.g., comparing with the WP on the null covariance structure;t=−5.04,p=.003andt=−5.06,p=.003, for the dense and sparse cases, respectively).

The comparison between the dense and sparse cases was of interest. Whereas the pairwise DCC-BL and the jointly trained DCC-J performed similarly for the*dense*case, DCC-BL performed better in the*sparse*case for the covariance structures with time-varying structure (e.g., comparing these two on the slow periodic covariance structure;t=−0.14,p=1.00, andt=9.66,$p=1.5\times 10^{-8}$, for the dense and sparse cases, respectively). The SW-CV estimates performed worse in the sparse case relative to the WP (e.g., on the fast periodic covariance structure;t=−1.46,p=1.00andt=−7.76,$p=3.8\times 10^{-7}$, for the dense and sparse cases, respectively). This suggests that it may also benefit from learning different parameters (window lengths in this case) for each edge, instead of a globally optimal window length.

### 3.2 Resting-state fMRI benchmarks

Under real-world conditions, the ground-truth covariance structure between brain components is unknown, making the assessment of TVFC estimation methods difficult. Next, we framed indirect prediction tasks as proxies to shed light on which method’s TVFC estimates were closer to the ground truth. We analyzed the time-varying covariance between ICA-identified networks for 812 subjects from the HCP. The estimates for several edges (i.e., covariances between two network time series) of a single scan of a single subject are shown inFigure 6. These estimates varied radically among the methods considered, underscoring the importance of the choice of estimation method. Estimates for training DCC in a joint (“-J”) or pairwise (“-BL”) manner strongly overlapped; therefore, the results for DCC-BL are omitted. The characteristics and differences in the estimates could be reasonably captured by three TVFC summary measures: mean, variance, and rate-of-change (Fig. 7). To illustrate, DCC-J estimates changed rapidly within a narrow range of correlation estimates, which was captured by the low variance and high rate-of-change.

**Fig. 6.**
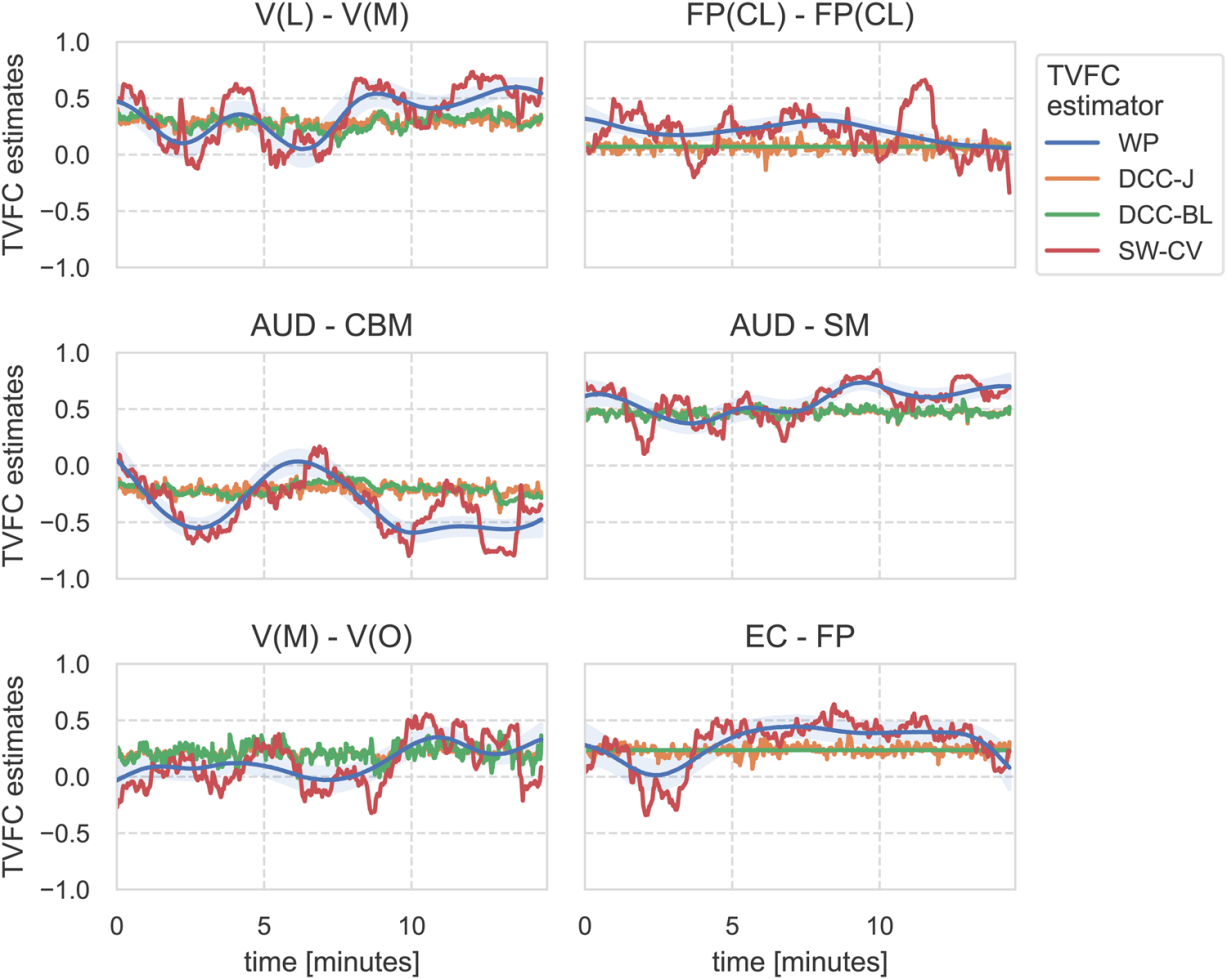
TVFC (correlation) estimates on ICA-identified time series from resting-state fMRI benchmark data from the Human Connectome Project, for a selection of edges for a single scan of a single subject. Estimates varied radically across methods, where Wishart process (WP) estimates resembled a smoothed version of the cross-validated sliding window (SW-CV) estimates. ICA components were mapped to functional networks: V, visual with lateral (L), medial (M), occipital (O) subsets; AUD, auditory; CBM, cerebellum; SM, sensorimotor; EC, executive control; FP, frontoparietal with cognition-language (CL) subset. DCC, dynamic conditional correlation, trained in joint (“-J”) and bivariate loop (“-BL”) fashion.

**Fig. 7.**
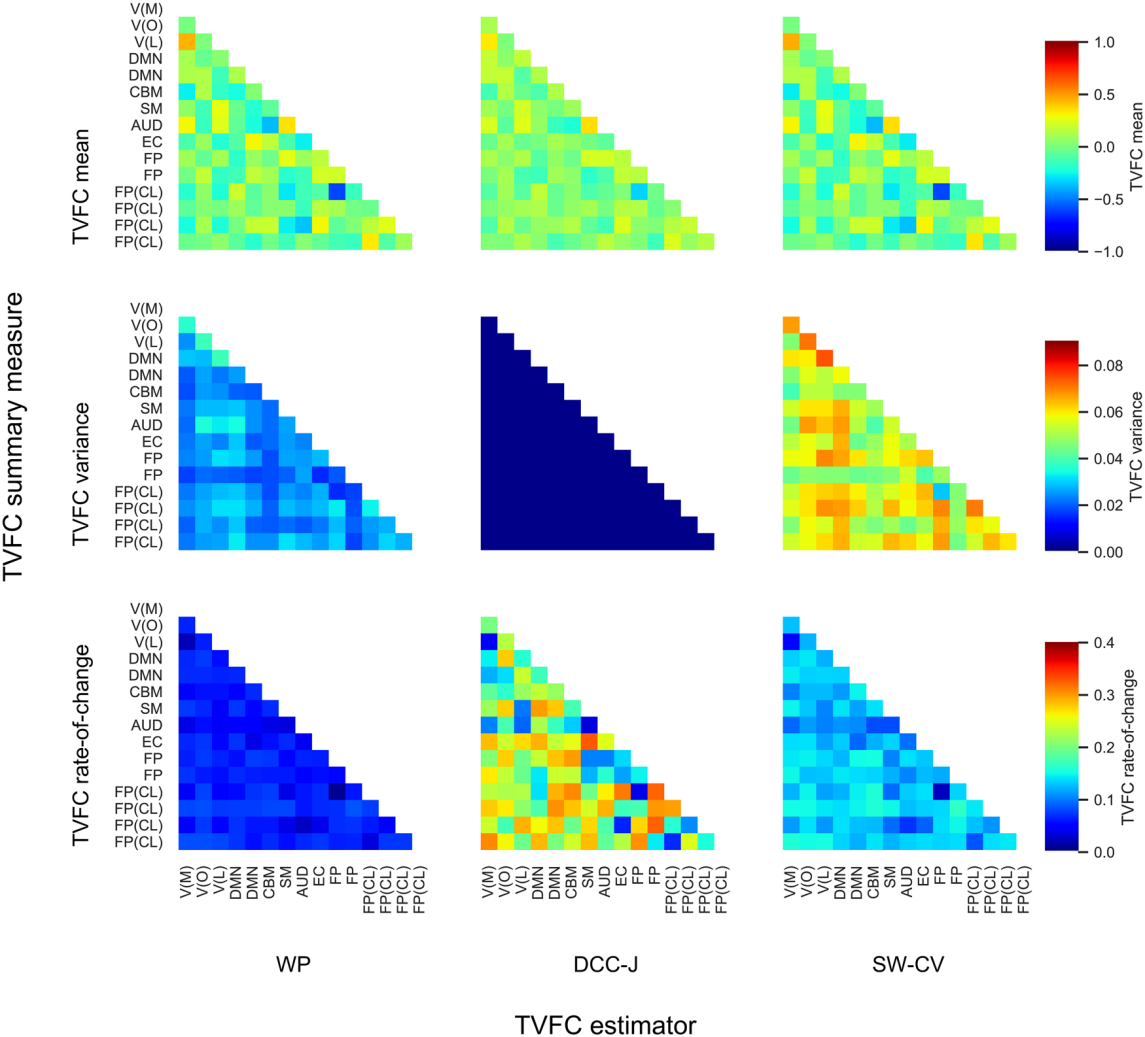
Edgewise TVFC summary measures for the first scan (1A) for resting-state fMRI benchmark data from the Human Connectome Project, averaged over 812 subjects. The three summary measures captured distinct characteristics of each TVFC estimation method. For interpretation, ICA components were mapped to functional networks: V, visual with medial (M), occipital (O), lateral (L) subsets; DMN, default mode network; CBM, cerebellum; SM, sensorimotor; AUD, auditory; EC, executive control; FP, frontoparietal with cognition-language (CL) subset. WP, Wishart process; DCC, dynamic conditional correlation, trained in joint (“-J”) fashion; SW-CV, cross-validated sliding windows.

**Fig. 8.**
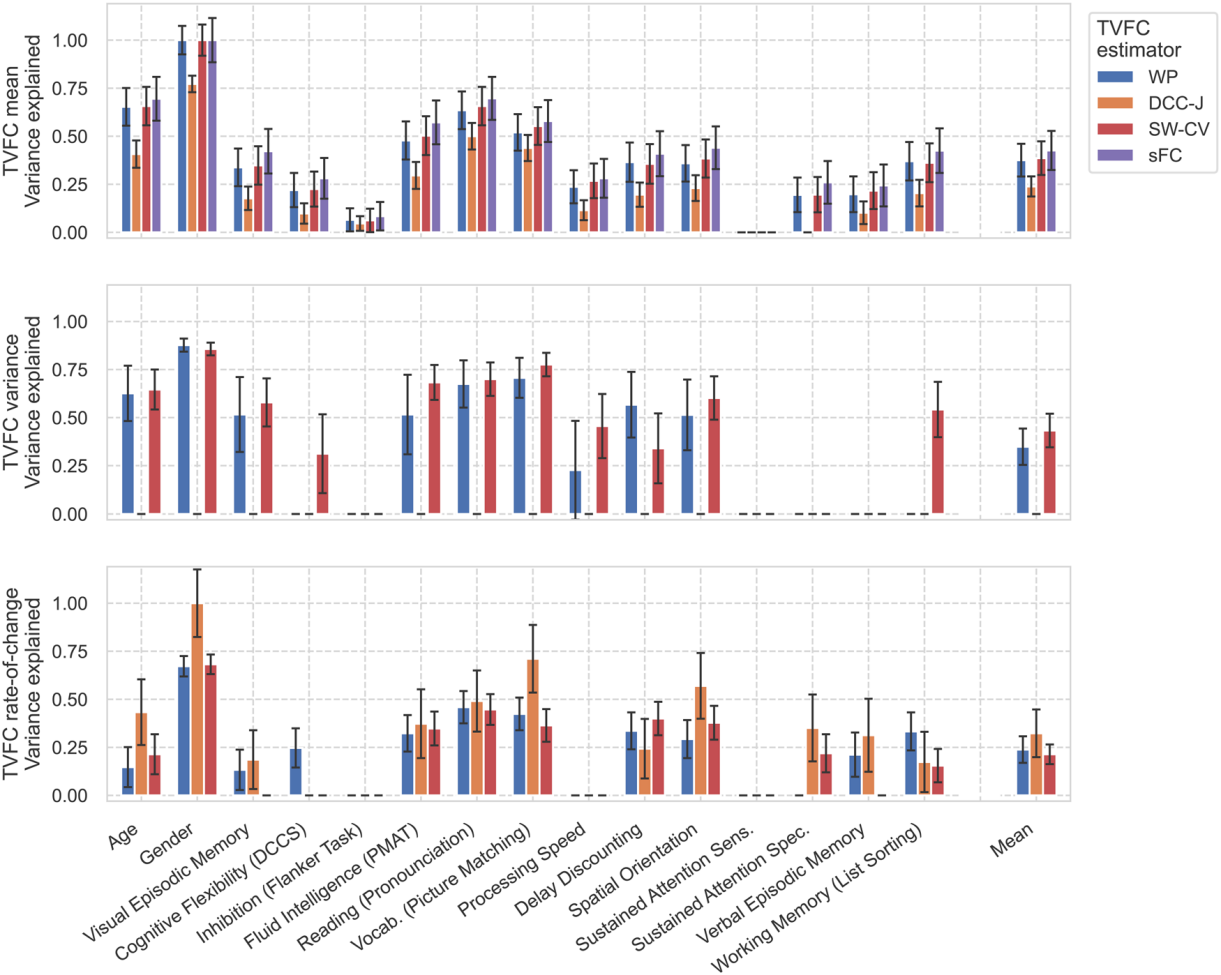
Subject measure prediction (morphometricity) scores for resting-state fMRI benchmark on data from the Human Connectome Project. The analysis was run separately for the TVFC summary measures of the mean (top), variance (middle), and rate-of-change (bottom row). Static functional connectivity (sFC) was added as a reference in the TVFC mean plot. The right-most column displays the mean across all subject measures. The error bars display the standard error, but the explained variance never exceeds one. Subject measures exhibited varying degrees of predictability from TVFC. Notable was the lack of predictive power of the variance of dynamic conditional correlation (DCC-J) estimates. A mapping between the interpretable item names printed here and the HCP-coded item names can be found in SupplementaryTable S1. WP, Wishart process; SW-CV, cross-validated sliding windows.

TVFC estimates were used to predict subject measures using a morphometricity analysis (Fig. 8) (Sabuncu et al., 2016). Strong heterogeneity was observed across the estimation methods and the predictive power of subject measures. For example, for the subject measure of Sustained Attention Sensitivity, none of the variance across all TVFC summary measures and estimation methods could be explained, indicating that the signatures of this task may not be captured by FC. Subject age was better predicted by the mean and variance of TVFC estimates than by the rate-of-change. To illustrate,Hutchison and Morton (2015)found that FC variability over the length of a scan correlates positively with age. We found that the WP and SW-CV methods, but not DCC-J, were predictive of age. The only significant differences in the average variance explained across all subject measures (the rightmost column inFig. 8) were the comparison between the mean of DCC-J estimates and the sFC estimates (t=−2.17,$p=3.8\times 10^{-2}$) and for the variance summary measure between WP and DCC-J (t=4.17,$p=2.7\times 10^{-4}$) and between DCC-J and SW-CV (t=−5.48,$p=1.0\times 10^{-5}$). This means that the WP and SW-CV methods exhibited similar performance. The complete lack of predictive power of the variance of DCC-J estimates was particularly noteworthy. Another popular way to frame this type of prediction task is as an out-of-sample predictive modeling study (Dhamala et al., 2021;Greene et al., 2018;Smith et al., 2015;Zamani Esfahlani et al., 2022). Such an analysis has been added toSupplementary Materials Section S.4for comparison. Our findings replicate those of prior studies, and the relative performance of the estimation methods is similar. However, DCC-J performed relatively better in the out-of-sample prediction task.

**Fig. 9.**
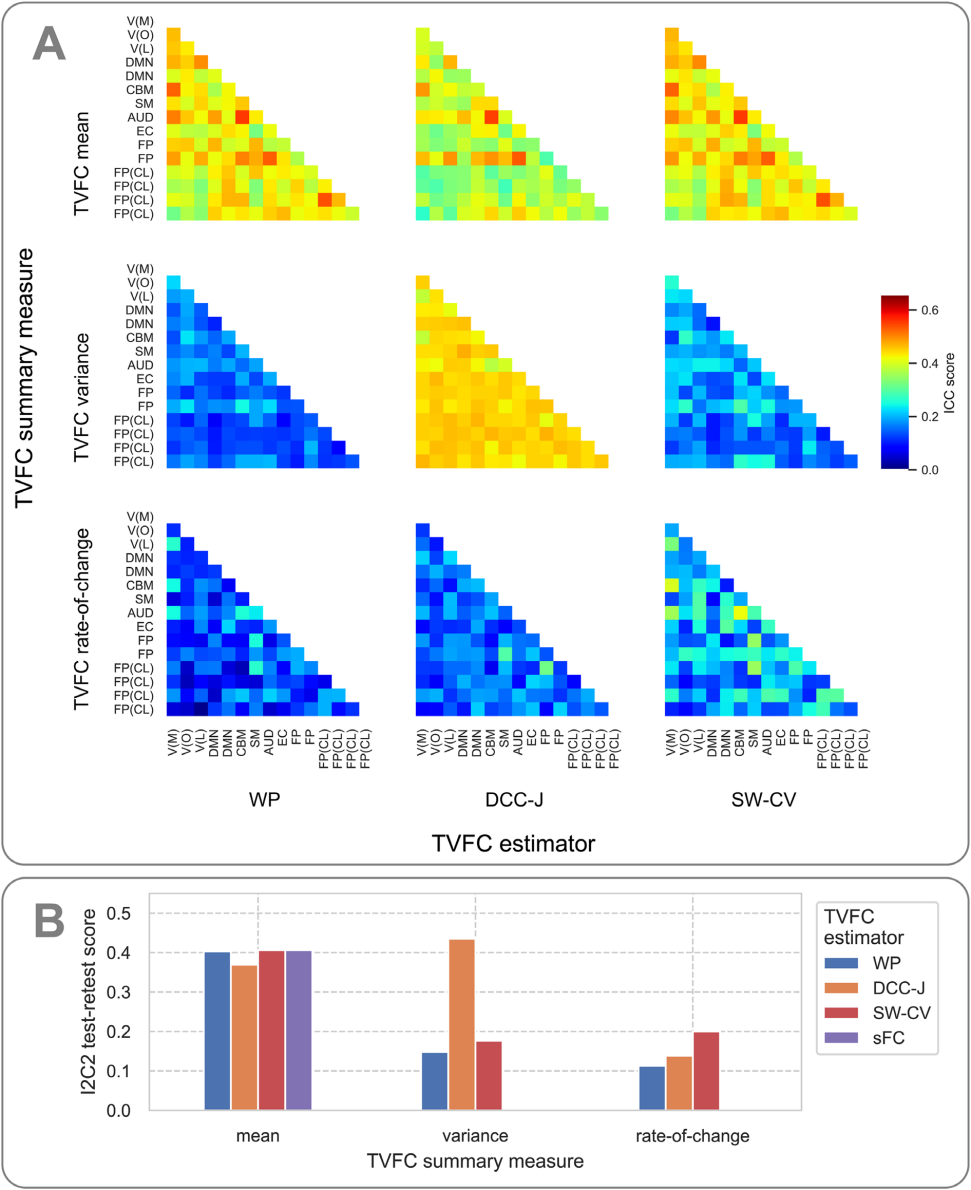
Test–retest robustness results for resting-state fMRI benchmark on ICA-identified data from the Human Connectome Project. (A) Edgewise ICC scores of estimated TVFC across four scans. Higher scores indicate a method’s ability to produce more robust subject-specific estimates. For interpretation, ICA components were mapped to functional networks: V, visual with medial (M), occipital (O), lateral (L) subsets; DMN, default mode network; CBM, cerebellum; SM, sensorimotor; AUD, auditory; EC, executive control; FP, frontoparietal with cognition-language (CL) subset. (B) Whole-brain test–retest results. I2C2 scores for mean, variance, and rate-of-change TVFC summary measures. WP, Wishart process; DCC, dynamic conditional correlation, trained in joint (“-J”) fashion; SW-CV, cross-validated sliding windows; sFC, static functional connectivity.

The test–retest benchmark examined the reliability of TVFC estimates across four scans of the same subjects, separately for each TVFC summary measure. The edgewise ICC scores are shown inFigure 9A. The mean of TVFC estimates was more robust than their dynamic summary measures (variance and rate-of-change). Some edges were consistently more robust than others, irrespective of the estimation method. The robustness of mean TVFC was similar across estimation methods (no significant differences were found), but the variance of DCC-J estimates was more robust compared with the other two methods (*t*-test between the mean ICC score over all edges,$p=4.63\times 10^{-17}$and$p=6.01\times 10^{-14}$, respectively, in comparison with the WP and SW-CV scores), replicatingChoe et al. (2017). This finding was perhaps unexpected, as the variance of DCC-J estimates was previously found to have no predictive power of subject measures. Further, the robustness of the rate-of-change of the SW-CV estimates was higher than that of the other two methods ($p=9.71\times 10^{-5}$and$p=1.18\times 10^{-2}$, in comparison with the WP and DCC-J scores, respectively). The I2C2 scores for whole-brain reliability of the TVFC summary measures were in broad agreement with the ICC scores (Fig. 9B). For perspective, the ICC scores for the learned WP kernel parameters were 0.29 for the kernel variance and 0.48 for the kernel length scales (Eq. 2.14).

Thek=3extracted brain states from the WP, DCC-J, and SW-CV estimates are shown inSupplementary Materials Section S.5. Apart from the extracted brain states, we were interested in the dynamics of the transitions between brain states. The number of brain state change points for each method for each subject is shown inFigure 10. The median number of brain state switches during a scanning session was one, zero, and nine for the WP, DCC-J, and SW-CV methods, respectively. Intuitively, given that SW-CV estimates have a higher rate-of-change (Fig. 7), it estimates more brain state switches than the WP.

**Fig. 10.**
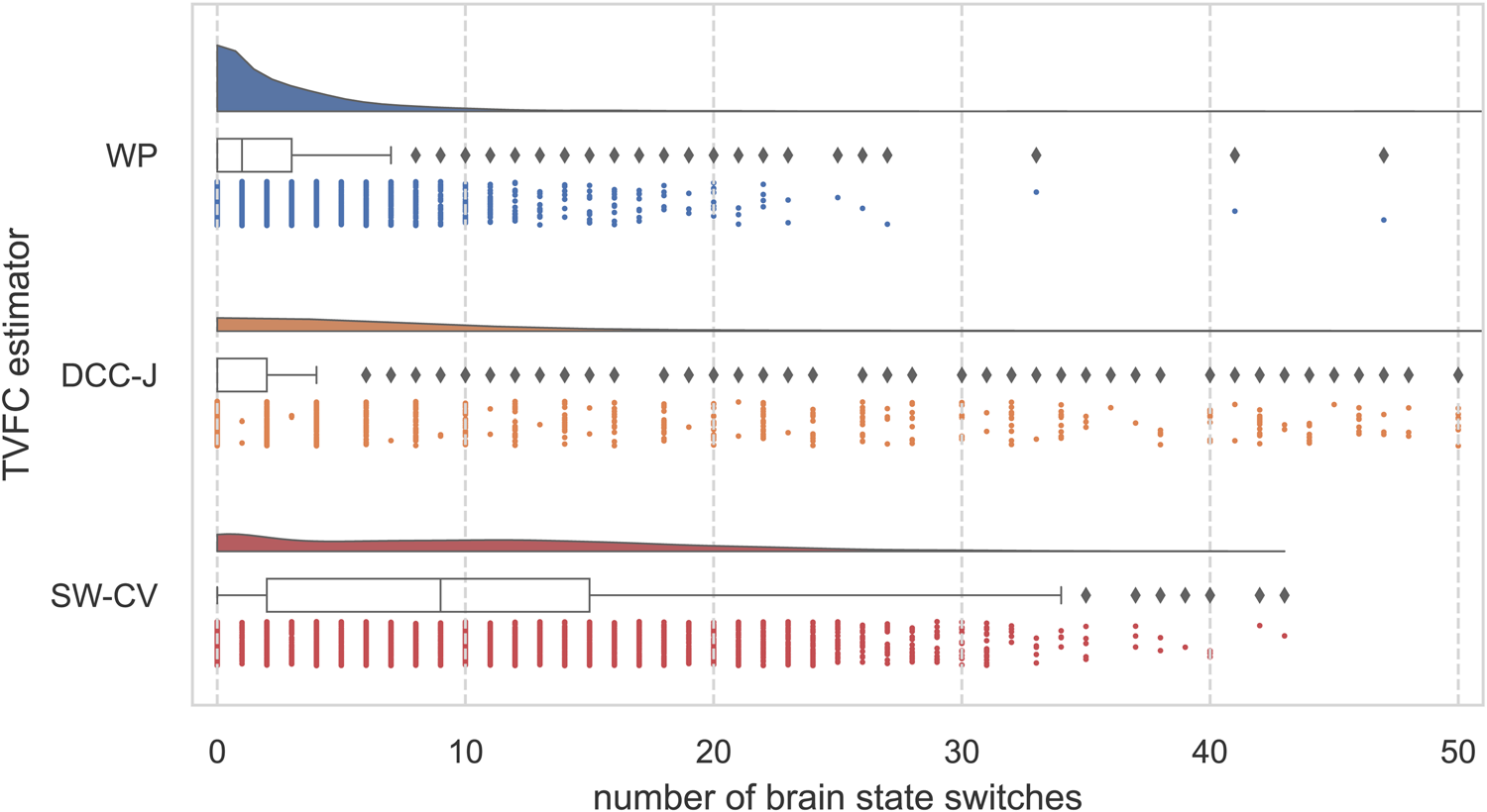
Brain state switch counts from brain state analysis of resting-state fMRI data from the Human Connectome Project. The raincloud plot of the number of brain state change points (switches between brain states) per TVFC estimation method includes the distribution of the number of switches; a boxplot showing the median, quartiles, and outliers; and a scatterplot below each boxplot representing individual participants. The total number of time points isN=1,200. WP, Wishart process; DCC, dynamic conditional correlation, trained in joint (“-J”) fashion; SW-CV, cross-validated sliding windows.

### 3.3 Task-based fMRI benchmarks

We used a tb-fMRI benchmark to investigate whether estimates of the dynamic coupling (TVFC) of V1 with five other brain regions could predict the presence of an external visual stimulus. Similar to the rs-fMRI case, ground-truth knowledge of the true underlying covariance structures was absent. However, in contrast to rs-fMRI, external stimulation can shape the coupling of regional brain activity in a more predictable and controllable manner. We observed (as expected) that the correlation between V1 and the other regions decreased as we moved up and away from the visual cortex hierarchy (Di et al., 2015): connectivity strength with V2 was the highest, followed by V3, V4, mPFC; and it was lowest with M1 (seeSupplementary Materials Section S.6).

**Fig. 11.**
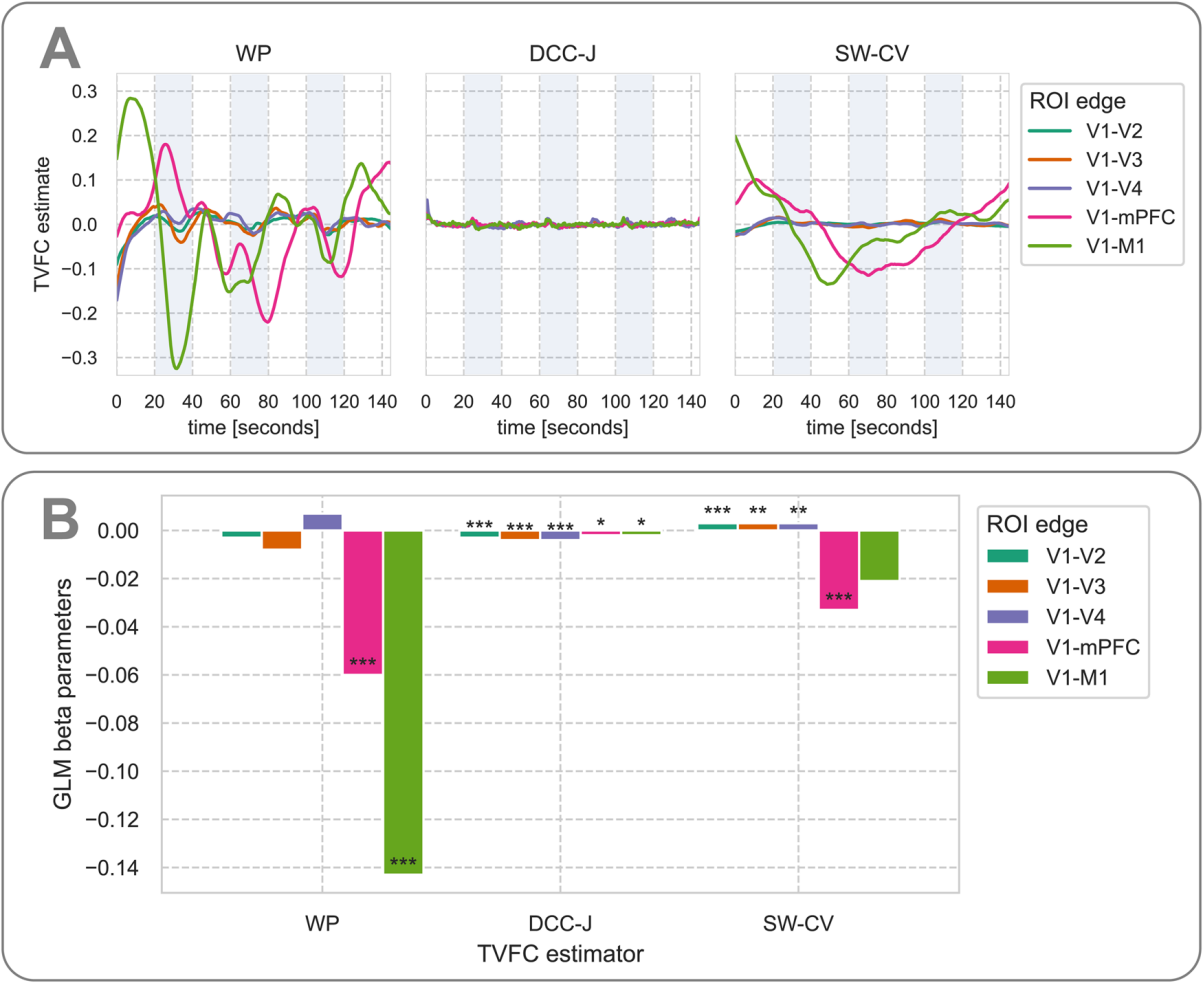
External stimulus prediction task-based fMRI benchmark results. (A) TVFC estimates for five edges between regions of interest (ROIs), averaged over 286 subjects from the Rockland dataset, after removing the (linear) trend and offset. Shaded areas indicate the presence of the external visual stimulus. (B) GLMβ(beta) parameters per TVFC estimation method, indicating the learned weights. Values farther from zero indicate that the GLM used the stimulus regressor more. Wishart process (WP) estimates were the most useful for predicting the presence of the external stimulus. Significance one-sided test*p*-values (after Bonferroni correction); *:p<.05, **:p<.01, ***:p<.001. DCC, dynamic conditional correlation, trained in joint (“-J”) fashion; SW-CV, cross-validated sliding windows; V1-V4, visual cortex regions; mPFC, medial prefrontal cortex; M1, primary motor cortex.

Next, as we expected that this external stimulus would induce periodic covariance between brain regions, we investigated the predictive utility of each covariance structure estimate for recovering the stimulus waveform. The relationship between the external stimulus and TVFC estimates is plotted inFigure 11, where the trend and offset were removed from the estimates and the estimates were averaged across subjects (Fig. 11A). The extractedβparameters from the GLM are shown to quantify this relationship (Fig. 11B). Most of the edges were anticorrelated to the external stimulus, as evidenced by the negative learned values. The WP model had the largest (absolute) estimated task-relatedβparameters and had thus captured most of the external task. The within-visual cortex edges had the least predictive power, and the V1-mPFC and V1-M1 edges had the most. This may indicate that connectivity between distant brain regions is more informative of external task conditions, which may be related to the stronger connectivity between visual regions (SupplementaryFig. S11B). Additional coupling effects in these edges may be more difficult to detect than in those with lower baseline connectivity.

### 3.4 Imputation benchmarks

A major difficulty in evaluating TVFC estimation methods is the lack of ground-truth knowledge of the covariance structures underlying observed time series. The imputation benchmark proposed here used observed time series directly as a ground truth to circumvent this issue. Data were split into equally sized training and test sets by removing every other scanning volume. Performance was assessed as the ability to predict unobserved test volumes from observed training volumes. Across the different datasets, we obtained results on this benchmark which corresponded to the performance metrics computed*with*knowledge of a ground truth or proxy thereof.

**Fig. 12.**
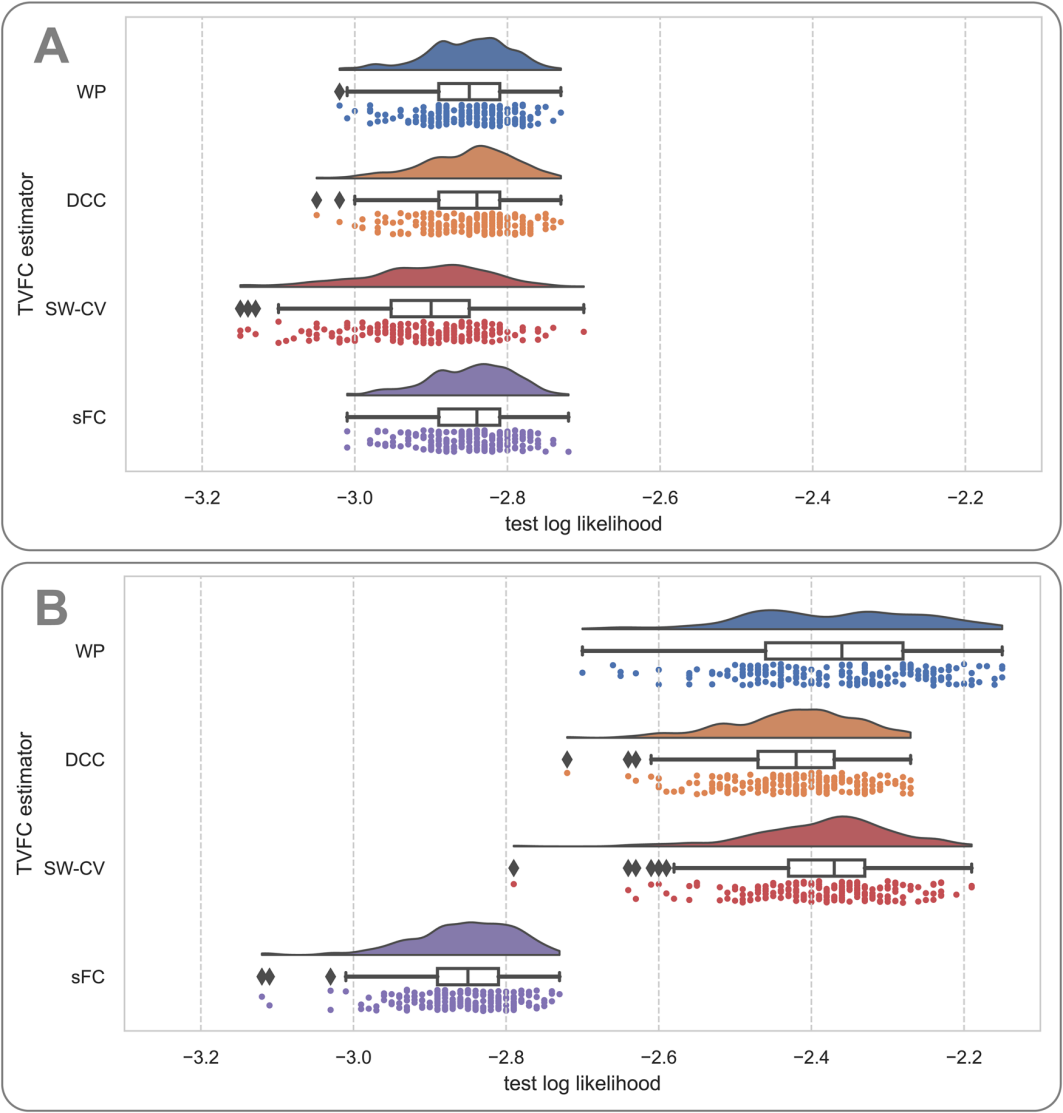
Imputation benchmark results for the simulated data. The raincloud plot of test log likelihoods for bivariate data forN=400time steps shows the distribution of test log likelihoods; a boxplot showing the median, quartiles, and outliers; and a scatterplot below each boxplot representing one of theT=200trials. Higher test likelihoods were preferred. (A) Null covariance. (B) Periodic (slow) covariance. WP, Wishart process; DCC, dynamic conditional correlation; SW-CV, cross-validated sliding windows; sFC, static functional connectivity.

For the simulated data, we examined two cardinal cases using bivariate noiseless data. For the null covariance case (Fig. 12A), all methods performed similarly well on this benchmark (e.g.,*t*-test between WP and sFC;p=.47). However, for a nonstatic covariance structure, such as the slowly oscillating periodic structure (Fig. 12B), we found that the sFC estimates performed much worse on the imputation benchmark than the other methods (e.g.,*t*-test between WP and sFC;$p=1.75\times 10^{-106}$). These findings correspond to the prior results shown for the simulation benchmarks, where the prespecified ground-truth covariance structure was reconstructed.

**Fig. 13.**
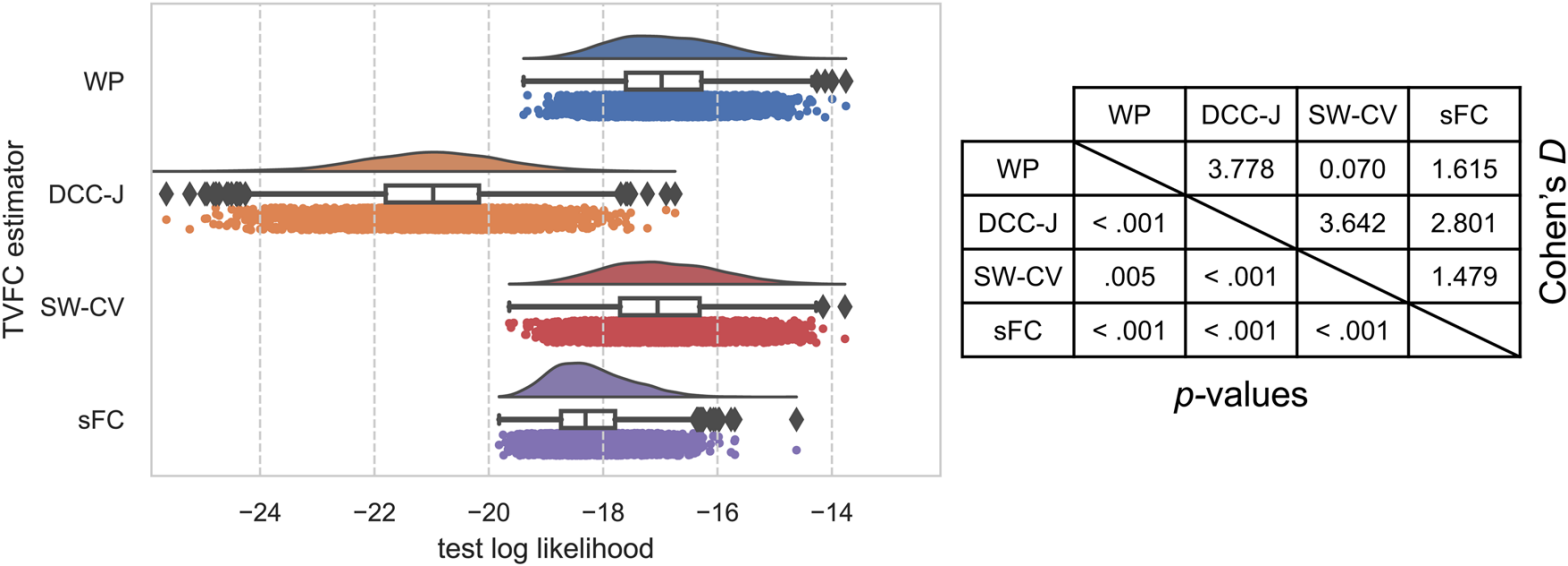
Imputation benchmark results on resting-state fMRI data from the Human Connectome Project. The raincloud plot of test log likelihoods, where higher test likelihoods are preferable, shows the distribution of test log likelihoods; a boxplot showing the median, quartiles, and outliers; and a scatterplot below each boxplot representing individual participants. The table on the right shows the effect sizes (Cohen’s*D*) and the*t*-test significance*p*-values. WP, Wishart process; DCC, dynamic conditional correlation, trained in joint (“-J”) fashion; SW-CV, cross-validated sliding windows; sFC, static functional connectivity.

Next, we ran the imputation benchmark on rs-fMRI data from the HCP. The WP and SW-CV methods outperformed DCC-J and sFC on the whole-brain (all edges) imputation benchmark (Fig. 13). DCC-J estimates performed worse than sFC estimates, indicating a poor model fit. This would underwrite the interpretation of the high test–retest scores for its variance as a result of numerical instability rather than being indicative of having modeled the data well.

There may be certain edges where WP performance was similar to that of the static approach (i.e., static edges), and some where it outperformed it (i.e., dynamic edges). Therefore, we also ran the imputation benchmark individually for each edge. These results (averaged over all subjects) are shown inFigure 14. Certain edges had high performance on this imputation benchmark, which may be because they were static and thus easier to fit. For example, test likelihoods were high for one of the FP(CL)-FP edges, which can be explained by the low variance and rate-of-change of this edge (seeFig. 7). However, we were interested in comparing estimation methods. The WP outperformed sFC more strongly for certain edges. This may be because these edges changed more over time, as indicated by the higher variance and rate-of-change summary measures for these edges (seeFig. 7).

**Fig. 14.**
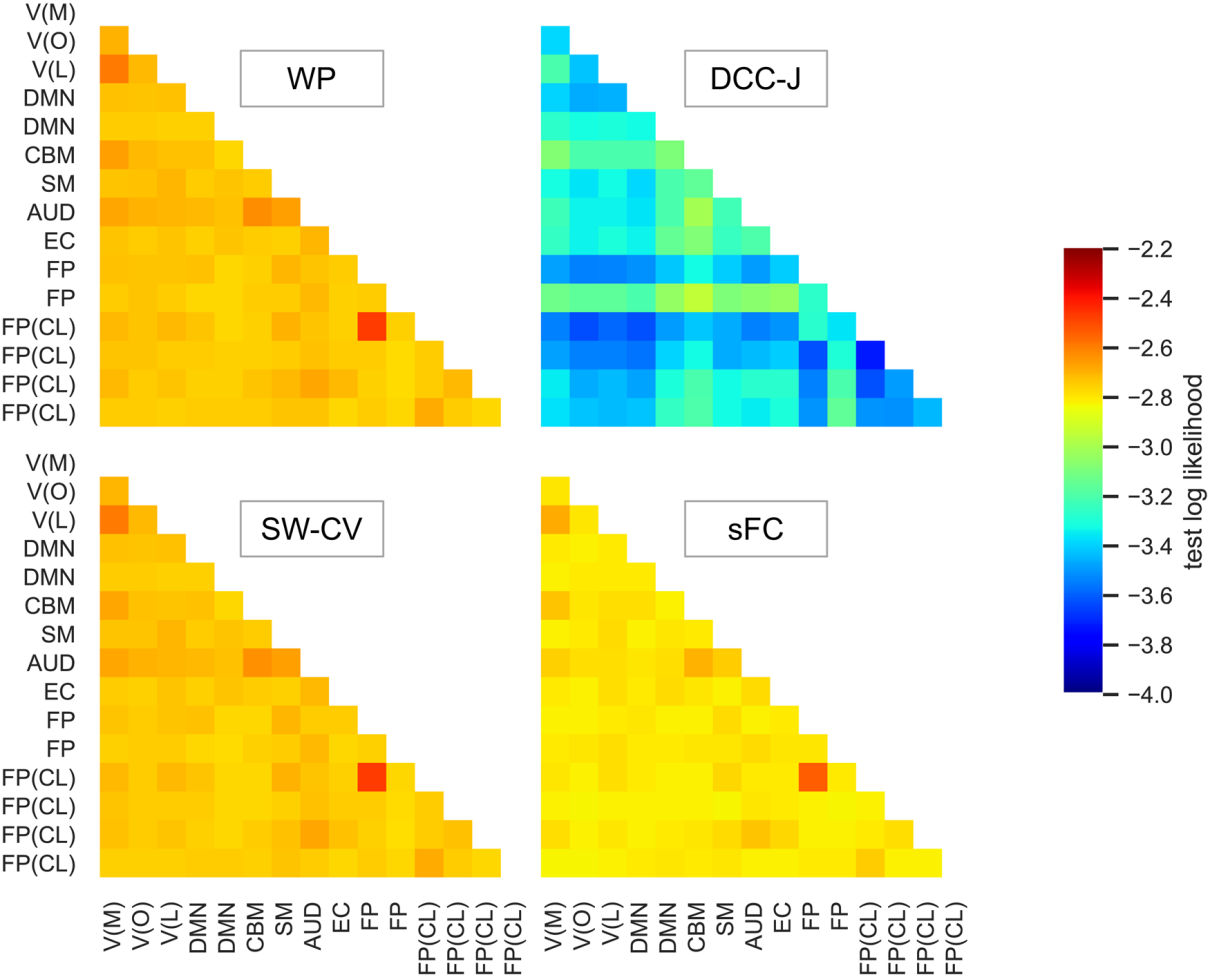
Imputation benchmark edgewise results on resting-state fMRI data from the Human Connectome Project. The equivalent of mean test log likelihoods fromFigure 13is shown for each edge individually. Higher test likelihoods were preferred. For interpretation, ICA components were mapped to functional networks: V, visual with medial (M), occipital (O), lateral (L) subsets; DMN, default mode network; CBM, cerebellum; SM, sensorimotor; AUD, auditory; EC, executive control; FP, frontoparietal with cognition-language (CL) subset. WP, Wishart process; DCC, dynamic conditional correlation, trained in joint (“-J”) fashion; SW-CV, cross-validated sliding windows; sFC, static functional connectivity.

**Fig. 15.**
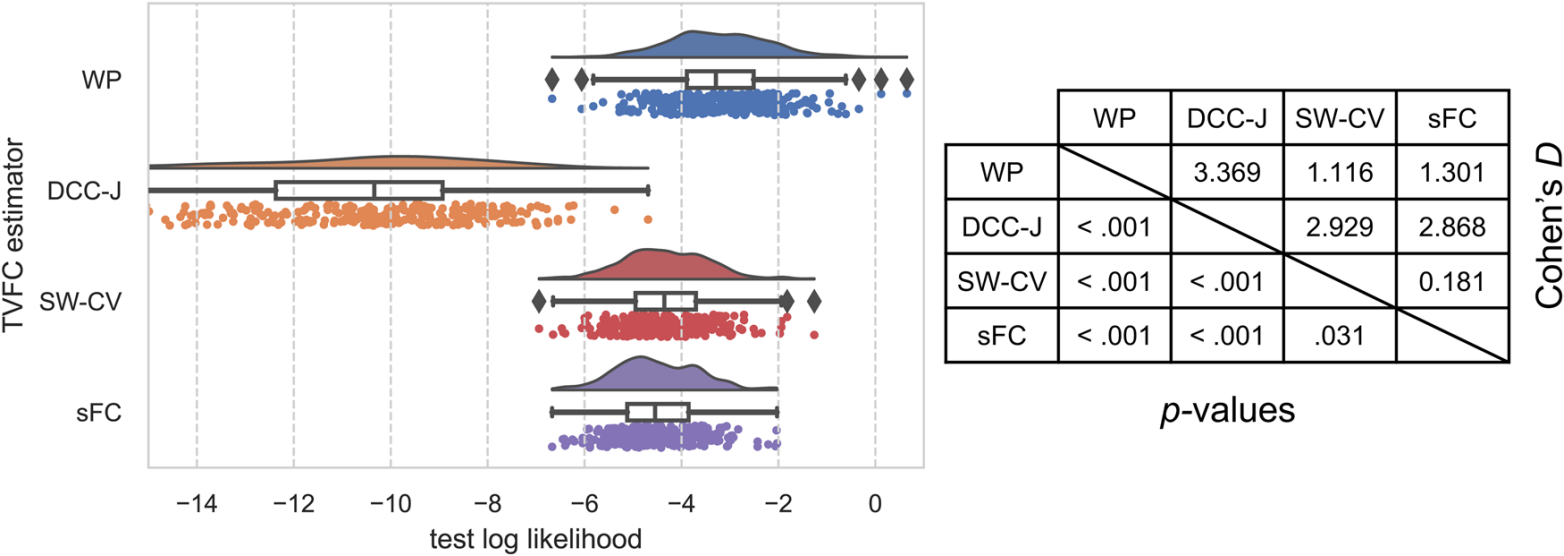
Imputation benchmark results for task-based fMRI Rockland data. The raincloud plot of test log likelihoods for 286 subjects, where higher test likelihoods were preferred, shows the distribution of test log likelihoods; a boxplot showing the median, quartiles, and outliers; and a scatterplot below each boxplot representing individual participants. The table on the right shows the effect sizes (Cohen’s*D*) and the*t*-test significance test*p*-values. WP, Wishart process; DCC, dynamic conditional correlation, trained in joint (“-J”) fashion; SW-CV, cross-validated sliding windows; sFC, static functional connectivity.

Applying the imputation benchmark to the tb-fMRI Rockland data showed strong performance for the WP and weak performance for DCC-J (Fig. 15). SW-CV outperformed sFC, but with a small effect size, indicative of its failure to capture the dynamics in these data.

## 4 Discussion

We investigated the utility of WPs as a natural method for TVFC estimation. Further, we introduced a data-driven method to determine the optimal window length in SW approaches. Method comparison and selection are intrinsically difficult because of the lack of access to the true covariance structure underlying neural activity. We proposed a range of prediction tasks to benchmark TVFC estimation methods and proposed a data-driven imputation benchmark. We found that the WP performed competitively.

We simulated BOLD time series representing putative covariance structures present in fMRI. We showed that the WP does not produce spurious estimates of covariance dynamics when the true covariance is static, while we replicated this well-known failure mode of the SW and DCC approaches (Lindquist et al., 2014;Lurie et al., 2020). This may be due to the WP’s strong prior over static covariance structures; its estimates will be static unless provided with sufficient evidence of the contrary. Further, SW methods estimate in a pairwise fashion and have less inherent protection against false positive estimation. They view each pair of time series independently from all other time series. The WP estimates outperformed the other methods in the case of a smoothly changing covariance structure. However, the WP was not particularly well suited for modeling covariance structures with sudden changes in connectivity strength. This could be attributed to the smoothness of the underlying GPs. The other methods considered (DCC and SW-CV) also failed in such cases. We found that the WP performed competitively when scaling to three dimensions, especially when the edges between the simulated time series had distinct covariance structures. Nonetheless, simulation studies have limited value as it remains unclear what covariance structures are present in real fMRI data.

We studied benchmarks using rs-fMRI data from the HCP. We found large qualitative differences in the TVFC estimates among the different estimation methods. We related summary measures of TVFC estimates with subject measures through a morphometricity analysis. The results for sFC (as a reference null model; the alternative hypothesis that FC is static) and the first summary measure (mean across time) of TVFC estimates replicated those of a previous study (Li, Kong, et al., 2019). The time-varying summary measures (variance and rate-of-change) contained information about subject measures, which justifies the study of TVFC beyond sFC. However, these simple summary measures may be limited characterizations of the dynamics of TVFC, and a more sophisticated mapping between TVFC estimates and subject measures may lead to an improved benchmark. WP and SW-CV estimates had the highest predictive power. However, they performed differently for different subject measures, which may indicate the capture of distinct elements of covariance structures. In terms of test–retest robustness, all methods performed similarly, but the variance of the DCC estimates and the rate-of-change of the SW-CV estimates were more robust across scanning sessions. However, the high robustness of the DCC estimates may be because the raw numerical values of the variance of the DCC estimates were close to zero. The test–retest measure may be unstable in cases where both within-subject and between-subject variances are small. Lastly, the brain state analysis demonstrated that the choice of TVFC estimation method impacts the number of brain state switches during a scan.

We used a tb-fMRI dataset from the Rockland sample with an external visual task that induced a covariance structure in the participants. The ability of covariance estimates to detect putative changes in interregion correlations induced by the stimulus was determined by regressing the stimulus onto the TVFC estimates. The WP estimates recovered the external stimulus better compared with the other methods, witnessed by learning larger weights for the stimulus in a GLM setup. This outperformance could be attributed to the small number of scan volumes. Bayesian methods generally perform well in small samples because they use priors, DCC is known to struggle in small samples, and SW-CV may not have seen enough data to find a reasonable window length, and is even more sensitive to outliers in such regimes. The highest predictive performance was found for the V1-M1 and V1-mPFC edges. V1 and M1 are anatomically farther apart than V1 and the other visual areas. They underlie functionally distinct processes, which may explain their anticorrelated interaction (Fox et al., 2005;Murphy et al., 2009). This may explain why the V1-M1 edge was most (anti)correlated with the external task conditions. It is possible that visual stimulus onset disrupts coactivity between V1 and M1 (Di et al., 2015). Further, Figure S11B indicates that the overall FC between V1 and the other visual areas was much stronger than with the mPFC and M1. The baseline strong static correlation between V1 and other visual areas may have masked any potential dynamic changes in connectivity induced by the stimulus. The same could be argued for the mPFC, potentially to a lesser degree. To investigate TVFC estimation beyond the simple external stimulus studied here, other tb-fMRI benchmarks with richer task structures should be included (Xie et al., 2019).

We found a correspondence between the estimation method performance on each benchmark (simulations, rs-fMRI, and tb-fMRI) and their respective performance on the respective imputation benchmarks. In other words, the WP performs well on this benchmark when it does so on the matched benchmark, and vice versa. This marks its exciting promise as a benchmark that can be used in realistic situations where no ground-truth covariance structure or proxies thereof (e.g., concurrent information, expert labeling, or additional collected participant information) are available.

Based on the simulation benchmark results, we posit that a method’s outperformance of the sFC estimate can be considered an indication of how time-varying the covariance structure is (but only if the method has successfully modeled the data). The TVFC estimation method performance was similar to that of sFC when either there was no or little dynamic structure present, or the methods failed to model it. However, the TVFC estimation methods outperformed sFC when there was a dynamic structure (if the method had successfully modeled it). In other words, we posit that outperformance over the sFC estimate is a proxy for a statistical test of whether there is any time-varying structure (Hindriks et al., 2016;Zalesky & Breakspear, 2015) and can be considered a null model study (Liégeois et al., 2021;Miller et al., 2018;Novelli & Razi, 2022). The imputation benchmark finding is consistent with this interpretation. Outperformance of the sFC estimate on this benchmark can also be considered a null model study. Therefore, practitioners are recommended to conduct this study prior to any further analysis, not only when in doubt about how to estimate TVFC, but also whether their data contain a strong dynamic component.

In addition to competitive benchmarking performance, the WP offers qualitative advantages, which may also explain its competitive performance. First, unlike SW, it is a*model-based*approach, which can lead to increased predictive power (Bassett et al., 2011), allowing for easier specification of inductive biases (Foti & Fox, 2019), and uncertainty modeling (Kudela et al., 2017). In general, a model that makes fewer assumptions about the data and extracts relevant hyperparameters automatically is preferred.

### 4.1 Limitations and future work

We must still consider the possibility of covariance structures in real data being organized as point processes of change points, where connectivity can suddenly and drastically change. All the considered models performed poorly on such data, for example, the simulated “state transition” dataset. State-based models such as HMMs are expected to perform better on such covariance structures. Change point detection algorithms can help determine whether such sudden changes exist (Cribben et al., 2012;Robinson et al., 2010). Within the WP framework, change point kernels can be included in models (Saatçi et al., 2010;Wilson, 2013). A key question is whether state transitions are expected in the covariance structures of real data. If methods that are better suited for detecting states outperform those that cannot capture change points well on real data benchmarks designed for this purpose, this would provide evidence for their existence.

Another limitation is that the utility of (rs-fMRI) test–retest studies is questionable. The classical measurement error framework assumes an immutable subject-specific property, whereas in FC studies, at least part of the functional architecture is expected to be different in different sessions, and thus less robust than more stable characteristics such as anatomical properties. One estimation method could extract subject-specific traits, such as participant age, whereas another could extract participant states, such as stress during the scan. It is not clear whether a TVFC estimation method that extracts stable subject- or session-specific information is preferred. This makes it a poorly framed exercise, and its interpretation in relation to the optimal method choice is unclear. Further, viewing this as a prediction problem, the test–retest problem can be stated as, when given the first scan, how well we can predict which scan is that subject’s subsequent scan. In this light, the method with the best test–retest score from*any*model feature can be considered stronger, such as the WP’s learned kernel parameters, for which we found high ICC scores. Although test–retest reliability may be desired, optimizing it for its own sake overlooks the goal of TVFC estimation. It has recently been argued that (behaviorally)*predictive*connectomes are more important than*reliable*connectomes (Finn & Rosenberg, 2021).

The WP implemented in this study can be augmented in several ways. First, one of the disadvantages of this model is its computational cost (Meng et al., 2023). To mitigate the computational cost due to large values ofN, innovations in sparse GPs that remove the need for inducing points can be ported to WP models (Adam et al., 2020;Wilkinson et al., 2021). However, this may be more relevant in applications to EEG data, where the number of time steps is much larger. The model scales in computational time with respect to*D*in$O(D^{3})$, owing to the required inversion of aD×Dmatrix. A low rank (or*factored*) implementation can help in situations whereDis large (Fox et al., 2015;Heaukulani & van der Wilk, 2019). This assigns each time series in a weighted manner toK≪Dclusters, and only learns the correlation structure between these clusters. Apart from reducing the number of model parameters, this can be considered as a dimensionality reduction step that posits an inherent network organization onto the brain. Second, while assuming the mean function$\mu_{n}$to be zero(s) is fair if we subtract the empirical mean from the data, models that include an estimate of the mean function may yield better covariance estimates (Lan et al., 2020). Finally, by specifying the similarity between observations, the kernel choice imposes assumptions (inductive bias) on the behavior of the observed time series, such as covariance structure smoothness (Foti & Fox, 2019;Fyshe et al., 2012). Expressive kernels can be designed to incorporate domain knowledge (Gönen & Alpaydin, 2011). For example, a periodic kernel could model TVFC where scanning volumes at a certain periodic interval are more related, which could be useful in the case of periodic task stimuli. Although our simple kernel choice in this work resembles a tapered SW approach, more complex kernels can introduce structures that are unattainable by other methods.

Having a comprehensive benchmarking framework is crucial because it allows for rapid iterative improvements and verification of model adjustments. Additional benchmarks will increase robustness (e.g., across data collection routines, scanners, and demographics) and deepen our knowledge of the utility of estimation methods. As a priority, a carefully tailored prediction task could help assess the degree to which brain states are artifacts of the estimation method choice or valid constructs that accurately describe underlying brain dynamics. Finally, we must accept that perfect benchmarking is unattainable in practice and that a single estimation method will not outperform in all configurations. One way to increase the robustness of our scientific conclusions is to formalize the TVFC estimation method choice (and other choices) using a multiverse analysis framework (Bell & Kampman, 2021;Steegen et al., 2016). Such approaches have recently been demonstrated in neuroimaging data processing pipeline choices (Dafflon et al., 2022) and for exploring machine learning model hyperparameter spaces (Bell et al., 2022). This views each decision “universe” in parallel and repeats an analysis in each universe. If a conclusion holds in most universes, it can be considered more robust.

## 5 Conclusions

This study assessed the utility of WP models for TVFC estimation. We also proposed a more principled method for running SW by cross-validating its window length. We carefully considered how to compare TVFC estimation methods by designing a comprehensive suite of benchmarks, including simulations, subject measure prediction, test–retest studies, brain state analyses, and external task prediction. A novel imputation benchmark based on cross-validation that can be run on any dataset was introduced. We showed how the WP performed competitively in relation to baselines, including both DCC and SW approaches, outperforming them especially on the external task prediction benchmark while estimating fewer false positives in the TVFC null model. Finally, we recommend running the imputation benchmark on any newly collected dataset to determine the optimal TVFC estimation method.

## Supplementary Material

Supplementary Material

## Data Availability

Researchers can use the pip-installable, open-source Python package*FCEst*(https://github.com/OnnoKampman/FCEst) for TVFC estimation using WPs and the baselines discussed. Code for reproducing the benchmarking and their results can be found athttps://github.com/OnnoKampman/FCEst-benchmarking. We used open-source Python libraries*nilearn*(Abraham et al., 2014),*pingouin*(Vallat, 2018), and*GPflow*(Matthews et al., 2017) and the*R*package*rmgarch*(Galanos, 2022). Human Connectome Project data are available athttps://db.humanconnectome.org/(requiring free registration). Rockland data are available athttp://fcon_1000.projects.nitrc.org/indi/pro/nki.html(requiring free registration).

## Appendix

### A.1. Updating ground-truth covariances after noise addition

Although the distribution of the HCP time series used as noiseϵin the simulation benchmarks is unknown, we know thatE[ϵ]=0and$E[\epsilon^{2}]=1$. First, we note that

$$E[{\text{y}}_{n}^{*}]=\alpha E[{\text{y}}_{n}]+\left(1-\alpha \right)E[\epsilon_{n}]=0,$$
(A. 1)

so that the variance of the new (noisy) time series is

$$\begin{array}{c} \sigma_{y_{n}^{*}}^{2}=E\left[\alpha^{2}{\text{y}}_{n}^{2}+2\alpha \left(1-\alpha \right){\text{y}}_{n}\epsilon_{n}+{\left(1-\alpha \right)}^{2}\epsilon_{n}^{2}\right]=\alpha^{2}+{\left(1-\alpha \right)}^{2}. \end{array}$$
(A. 2)

Regarding the covariance terms, all cross-terms except within${\text{y}}_{n}$are zero; therefore, we are simply left with$\sigma_{y_{n,1}^{*}y_{n,2}^{*}}=\alpha^{2}\sigma_{y_{n,1}y_{n,2}}$. To maintain unit time series variances, the new (noisy) signal is

$${\text{y}}_{n}^{*}=\frac{1}{\sqrt{\alpha^{2}+{\left(1-\alpha \right)}^{2}}}\left[\alpha \;{\text{y}}_{n}+\left(1-\alpha \right)\epsilon_{n}\right],$$
(A. 3)

so our new ground-truth covariance terms can be analytically updated as

$$\sigma_{y_{n,1}^{*}y_{n,2}^{*}}=\frac{\alpha^{2}}{\alpha^{2}+{\left(1-\alpha \right)}^{2}}\sigma_{y_{n,1}y_{n,2}}.$$
(A. 4)

### A.2. Human connectome project (HCP) data description

HCP participants were young adults (twins and nontwin siblings), aged 22–35 years. We used the data from the HCP S1200 public release (published in March 2017), from the 812 subjects with four available scans processed using improved fMRI image reconstruction (“recon2”). These scans were acquired with a 3T Siemens connectome-Skyra using a multiband sequence. The readily availableD=15dimensional ICA-based node time series were used. For interpretability, each ICA component was mapped to a well-known functional network (FN) (Giorgio et al., 2018). Three neuroimaging researchers visually inspected and manually assigned each ICA component to 1 of 10 networks that resemble task-driven FNs (Fox & Raichle, 2007;Laird et al., 2011;Smith et al., 2009). The four scans were divided into two immediately consecutive scans on two separate days (scans 1A, 1B, 2A, and 2B). Scans were acquired with a TR of 0.72 seconds and a voxel size of 2 mm isotropic. Each scan containedN=1,200images and was 15 minutes long. The data had been preprocessed using a (minimal) preprocessing pipeline (Glasser et al., 2013,^2016^;Smith, Vidaurre, et al., 2013). This minimal preprocessing pipeline included motion and distortion correction, and anatomical registration to standard MNI space. Noise components had been filtered out during preprocessing using ICA+FIX (Griffanti et al., 2014;Salimi-Khorshidi et al., 2014). Further global signal regression was not performed. All time series had been demeaned, with variance normalized (Beckmann & Smith, 2004).

### A.3. Rockland data description

The Rockland dataset contained fMRI scans of subjects (aged 18-35 years) alternating between fixating in the dark or seeing a checkerboard visual pattern consecutively (20 seconds in duration of both stimuli and rest periods). We used the dataset with a TR of 0.645 seconds (N=240). We only used data from the first baseline visit (coded as “ses-BAS1”). Data preprocessing was performed in FSL (Jenkinson et al., 2012) consisting of brain extraction and field-of-view fixation, data registration, motion correction, smoothing (5 mm Gaussian kernel), and detrending (high-pass, 0.01 Hz). No initial volumes were excluded from the scans. Finally, we denoised the data using ICA-AROMA (Pruim et al., 2015). The data were not prewhitened to ensure preservation of the visual task. Subjects with a correlation between their V1 BOLD time series and the stimulus time series (convolved with an HRF) of less than 0.4 were discarded, resulting in 286 subjects. Such low correlations indicated that the expected BOLD signature in V1 was not observed (e.g., owing to the subjects closing their eyes). This visual task is simpler than those recorded under the umbrella of the HCP, which makes it better suited for our benchmarking aims (Barch et al., 2013). We extracted BOLD activations from visual areas V1, V2, V3, V4, the medial prefrontal cortex (mPFC), and the primary motor cortex (M1). A plot of the time series, aligned across subjects, is shown inFigure S11A. As motivation for using this design,Di et al. (2015)showed that FC is not static across such visual stimuli and that FC between higher and lower visual regions is affected in a consistent manner with stimulus onset and offset.
