# Supplementary material for: Time-varying functional connectivity as Wishart processes
Source: Imaging Neurosci (Camb). 2024 Jun 5;2:imag-2-00184. doi: 10.1162/imag_a_00184 (PMC12290605; doi:10.1162/imag_a_00184)
Supplement: Supplementary Material [file imag_a_00184-supp.pdf]

SUPPLEMENTARY MATERIALS

S.1 Cross-validating sliding window length

The empirical validation of the effectiveness of the cross-validating SW window lengths is shown in Fig. S1. The cross-validated sliding windows (SW-CV) approach performed competitively for each synthetic covariance structure by adapting its window length to the covariance structure. A short prespecified window length (e.g., SW-30) performed poorly on the static **null** and **constant** covariance structures, but performed well on dynamic covariance structures such as the **periodic (fast)** and **boxcar** structures. A long prespecified window (e.g., SW-120) performed in opposite fashion: good performance on these static covariance structures and poor performance on the dynamic ones. Cross-validating the window length ensured that SW performed well in both static and dynamic domains. This reduces the need for an a priori hypothesis regarding the nature of the correlation structure in a certain dataset.

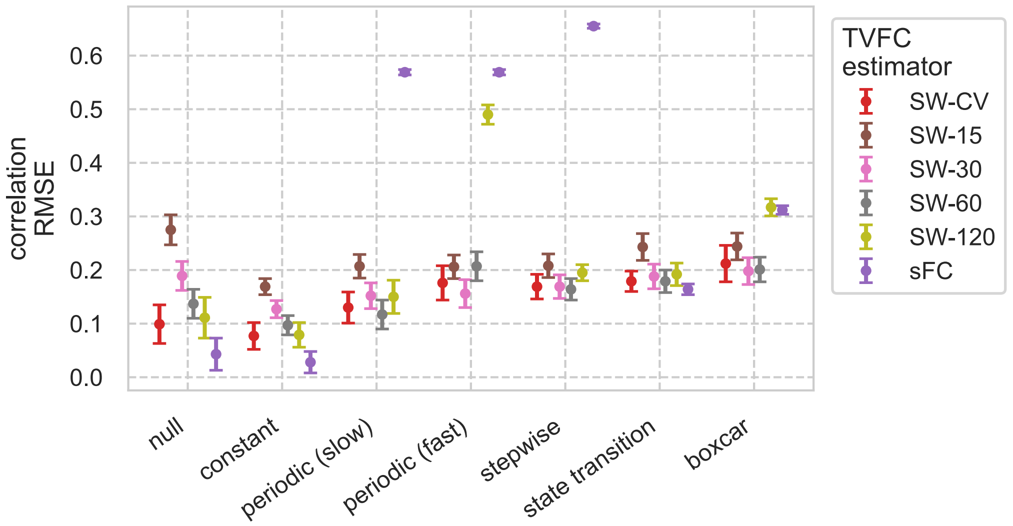

Fig. S1. Simulation benchmarks bivariate quantitative results comparing cross-validated sliding windows (SW-CV) to a range of SW models with fixed, prespecified lengths (denoted as the number of data points within the respective windows; SW-15 had 15 data points in its window). Performance was quantified as the root mean squared error (RMSE) between TVFC estimates and ground truth for all prespecified covariance structures with added rs-fMRI noise (SNR of 2) for $N = 400$ time steps. Means and standard deviations are shown across $T = 200$ trials. Abbreviations: sFC; static functional connectivity.

S.2 Higher dimensional simulation studies

In realistic settings, researchers work with datasets in which the number of time series is greater than two or three, as studied in the simulation benchmarks. Therefore, we scaled up the sparse trivariate case to $D = \{4, 6, 9, 15\}$ by adding additional

uncorrelated time series. The quantitative results for these datasets are shown in Figs. S2-S5. Detailed results from statistical tests between methods can be found in the code base of this paper at https://github.com/OnnoKampman/FCEst-benchmarking.

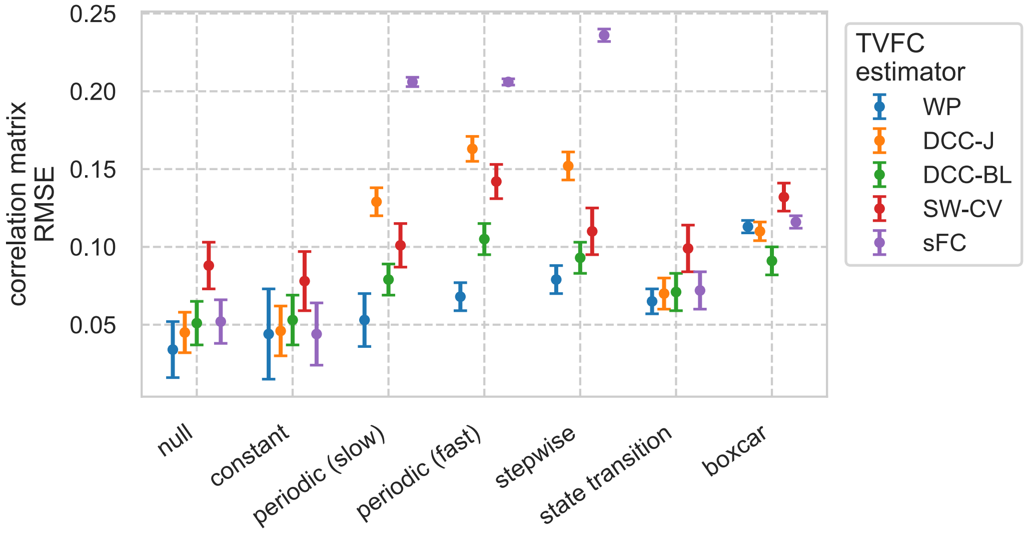

Fig. S2. Simulation benchmarks sparse four-dimensional quantitative results. Performance was quantified as the root mean squared error (RMSE) between TVFC estimates and ground truth for all prespecified covariance structures with added rs-fMRI noise (SNR of 2) for $N = 400$ time steps. Means and standard deviations are shown across $T = 10$ trials. Abbreviations: WP; Wishart process, DCC; dynamic conditional correlation, trained in joint ('-J') and bivariate loop ('-BL') fashion, SW-CV; cross-validated sliding windows, sFC; static functional connectivity.

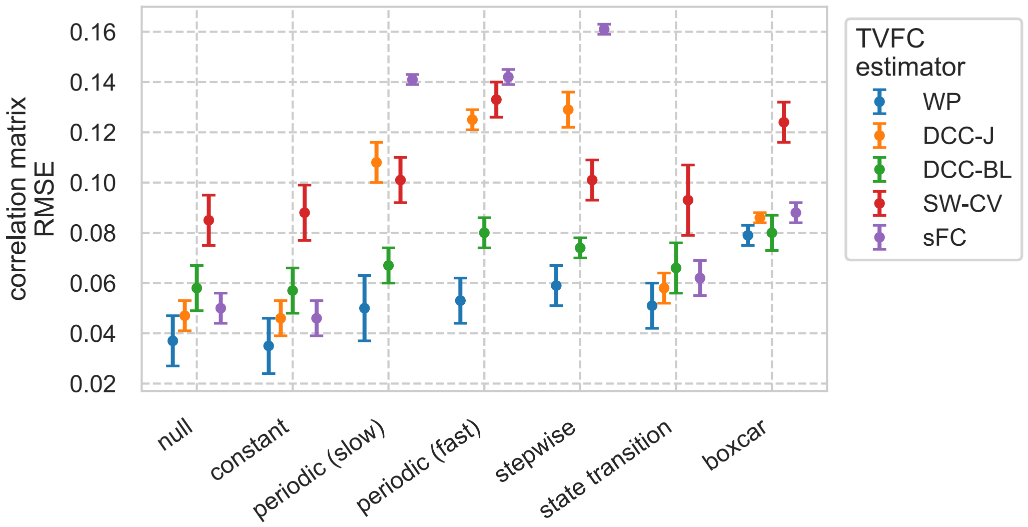

Fig. S3. Simulation benchmarks sparse six-dimensional quantitative results. Performance was quantified as the root mean squared error (RMSE) between TVFC estimates and ground truth for all prespecified covariance structures with added rs-fMRI noise (SNR of 2) for $N = 400$ time steps. Means and standard deviations are shown across $T = 10$ trials. Abbreviations: WP; Wishart process, DCC; dynamic conditional correlation, trained in joint ('-J') and bivariate loop ('-BL') fashion, SW-CV; cross-validated sliding windows, sFC; static functional connectivity.

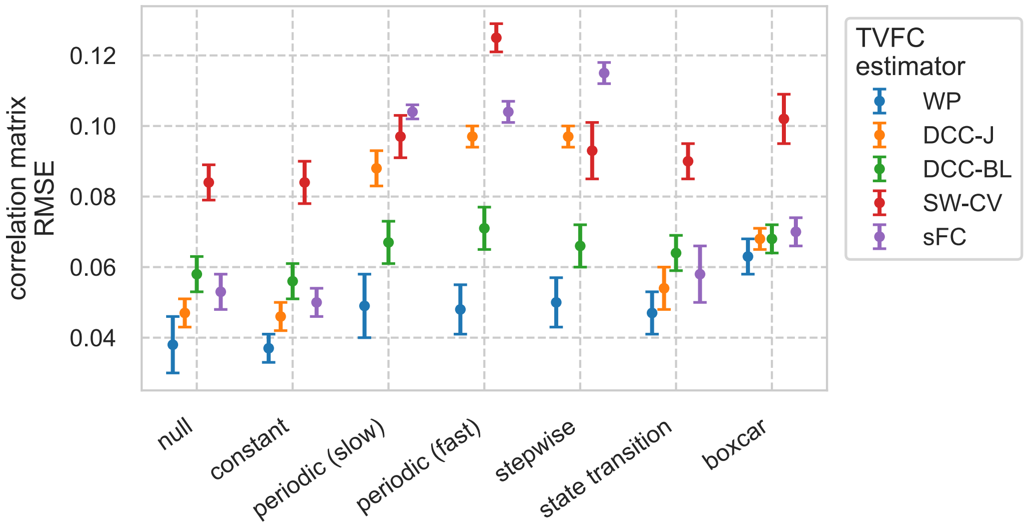

Fig. S4. Simulation benchmarks sparse nine-dimensional quantitative results. Performance was quantified as the root mean squared error (RMSE) between TVFC estimates and ground truth for all prespecified covariance structures with added rs-fMRI noise (SNR of 2) for $N = 400$ time steps. Means and standard deviations are shown across $T = 10$ trials. Abbreviations: WP; Wishart process, DCC; dynamic conditional correlation, trained in joint ('-J') and bivariate loop ('-BL') fashion, SW-CV; cross-validated sliding windows, sFC; static functional connectivity.

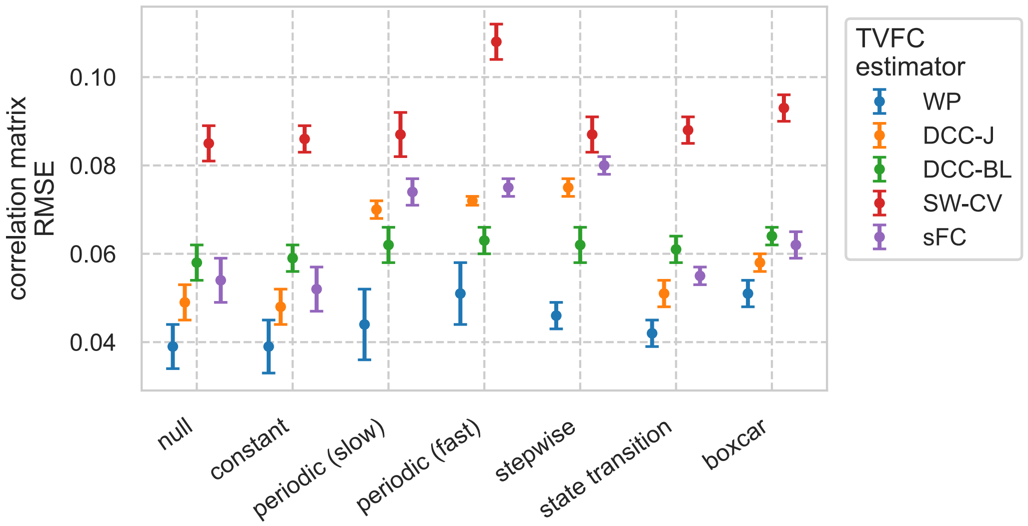

Fig. S5. Simulation benchmarks sparse fifteen-dimensional quantitative results. Performance was quantified as the root mean squared error (RMSE) between TVFC estimates and ground truth for all prespecified covariance structures with added rs-fMRI noise (SNR of 2) for $N = 400$ time steps. Means and standard deviations are shown across $T = 10$ trials. Abbreviations: WP; Wishart process, DCC; dynamic conditional correlation, trained in joint ('-J') and bivariate loop ('-BL') fashion, SW-CV; cross-validated sliding windows, sFC; static functional connectivity.

S.3 HCP items coding

A mapping between the coded HCP items (Van Essen et al., 2012, 2013) used in this study and the more interpretable names printed in the main paper (Fig. 8) is shown in Tab. S1. Cognitive scores were obtained using the NIH Toolbox Cognition Battery (Heaton et al., 2014), which assessed five cognitive domains: executive function, episodic memory, working memory, processing speed, and language.

Tab. S1. HCP item mapping to interpretable names used in the main paper. All items were classified as cognitive items. Interpretable item names were copied from Li et al. (2019). Not all items were assigned to a cognitive domain.

**HCP item code**	**Interpretable item name**	**Cognitive domain**
PicSeq_Unadj	Visual Episodic Memory	Episodic memory
CardSort_Unadj	Cognitive Flexibility (DCCS)	Executive function
Flanker_Unadj	Inhibition (Flanker Test)	Executive function
PMAT24_A_CR	Fluid Intelligence (PMAT)	
ReadEng_Unadj	Reading (Pronounciation)	Language
PicVocab_Unadj	Vocab. (Picture Matching)	Language
ProcSpeed_Unadj	Processing Speed	Processing speed
DDisc_AUC_40K	Delay Discounting	
VSPLOT_TC	Spatial Orientation	
SCPT_SEN	Sustained Attention (Sens.)	
SCPT_SPEC	Sustained Attention (Spec.)	
IWRD_TOT	Verbal Episodic Memory	Episodic memory
ListSort_Unadj	Working Memory (List Sorting)	Working memory

S.4 HCP out-of-sample prediction task

In the main paper, we related TVFC estimates to subject measures through a morphometricity analysis. Another common way to relate estimated TVFC to subject measures is to structure it as an out-of-sample prediction task (Dhamala et al., 2021; Greene et al., 2018; Smith et al., 2015; Zamani Esfahlani et al., 2022).

For comparison, we replicated an out-of-sample prediction task using linear ridge regression, which is a linear model with an L2 regularization term (Dhamala et al., 2021). We related the same vectors of summary measures of TVFC estimates to each subject measure individually, where TVFC estimates were averaged across the four scanning sessions. The weight of the regularization term was cross-validated. This model was implemented using Scikit-learn (Pedregosa et al., 2011). No nuisance variables, such as age (Damoiseaux, 2017) and gender (Weis et al., 2020), as used in the morphometricity model, were included. The model was trained over 100 permutations with a train/test split of 80/20, individually for each subject measure. The results of this analysis are shown in Figs. S6 (R2 scores) and S7 (prediction accuracy).

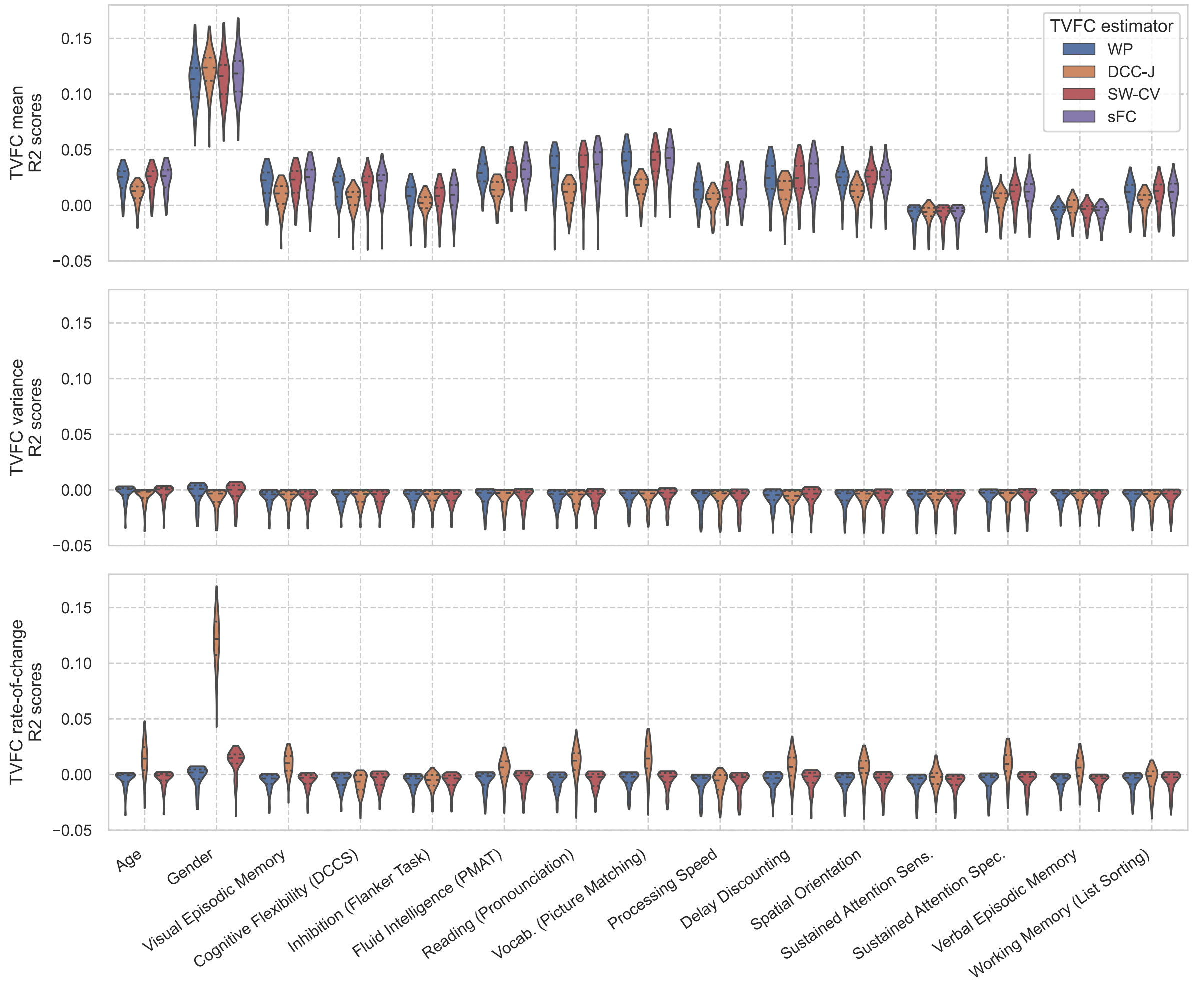

Fig. S6. Out-of-sample prediction task R2 scores for TVFC estimation methods considered for Human Connectome Project (HCP) subject measures. The dashed lines within each violin plot indicate the quartiles of the score distribution. Abbreviations: WP; Wishart process, DCC; dynamic conditional correlation, trained in joint ('-J') fashion, SW-CV; cross-validated sliding windows, sFC; static functional connectivity.

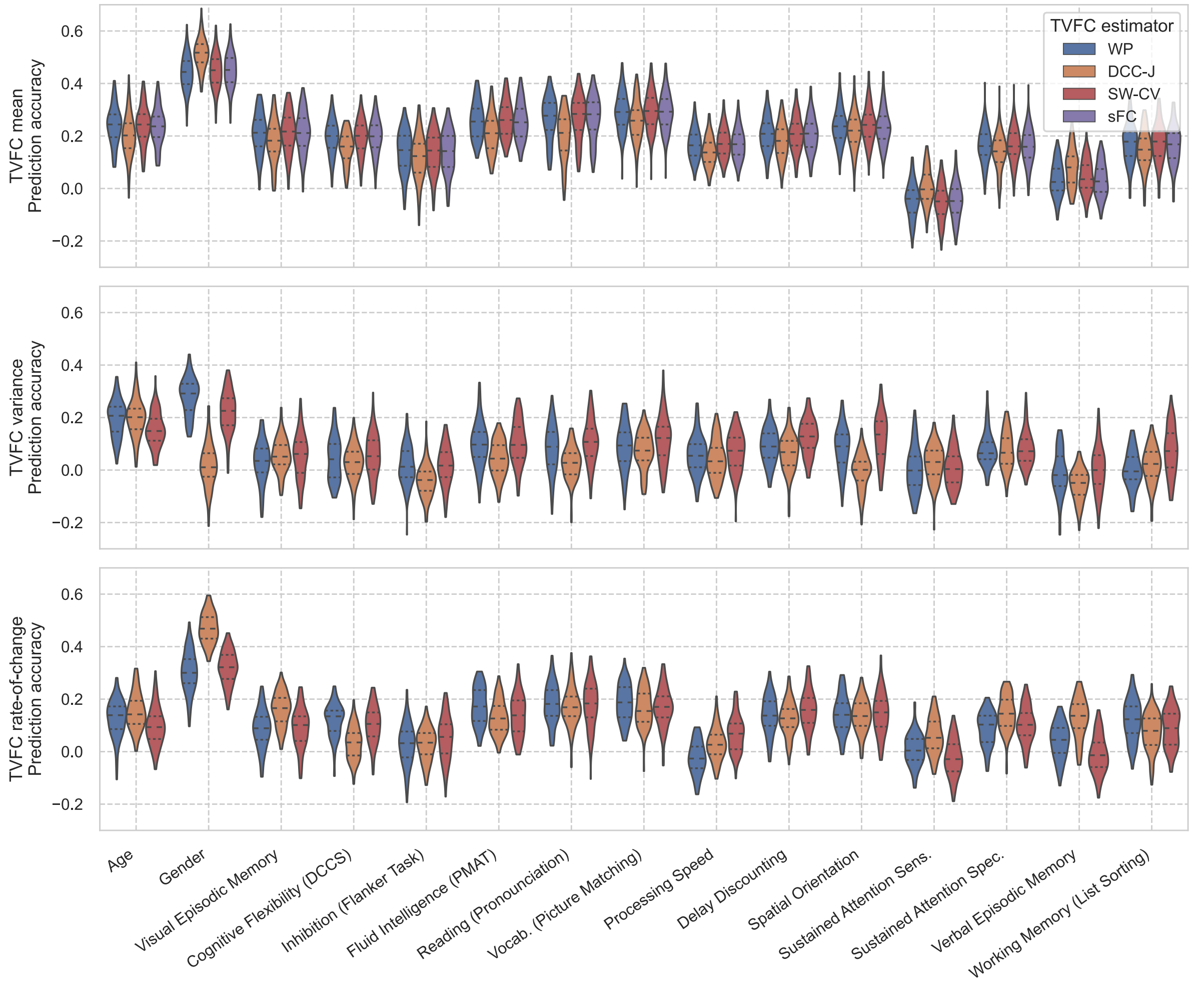

Fig. S7. Out-of-sample prediction task accuracy (defined as the correlation between model estimates and true values) for TVFC estimation methods considered for Human Connectome Project (HCP) subject measures. The dashed lines within each violin plot indicate the quartiles of the score distribution. Abbreviations: WP; Wishart process, DCC; dynamic conditional correlation, trained in joint ('-J') fashion, SW-CV; cross-validated sliding windows, sFC; static functional connectivity.

S.5 Extracted brain states

The brain states extracted from the TVFC estimates are shown in Figs. S8-S10.

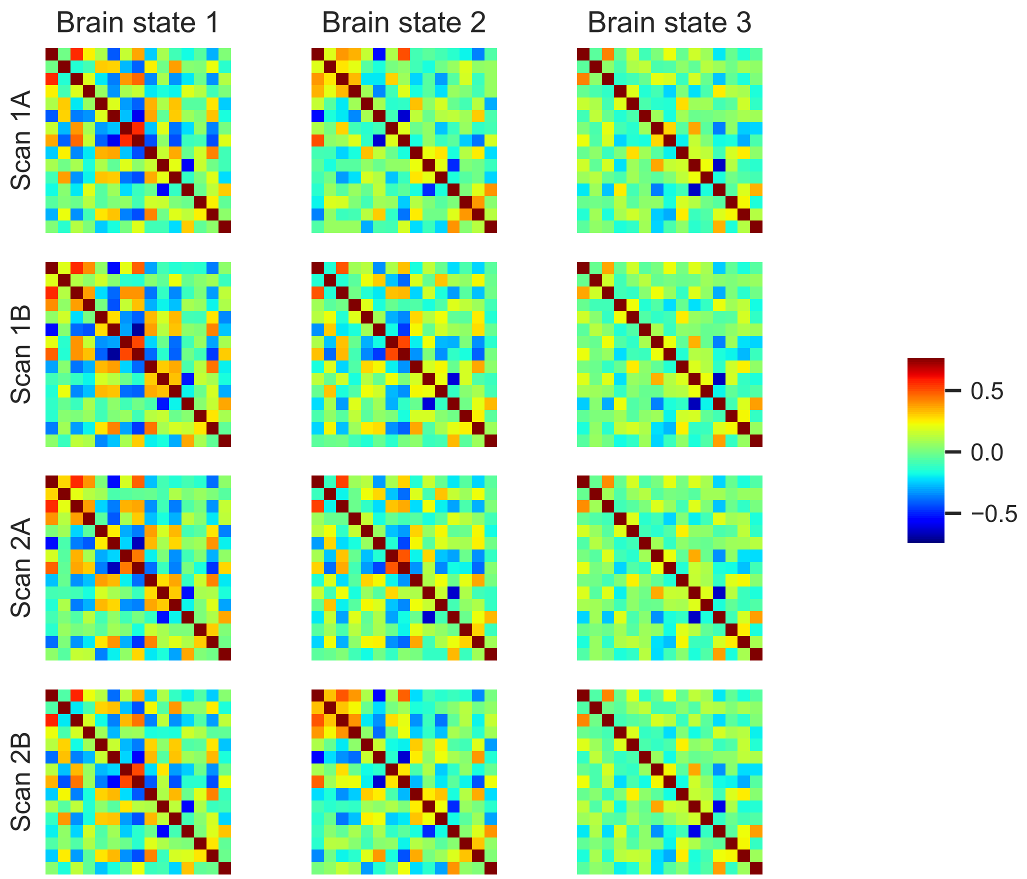

Fig. S8. Human Connectome Project (HCP) extracted brain states from Wishart process (WP) estimates.

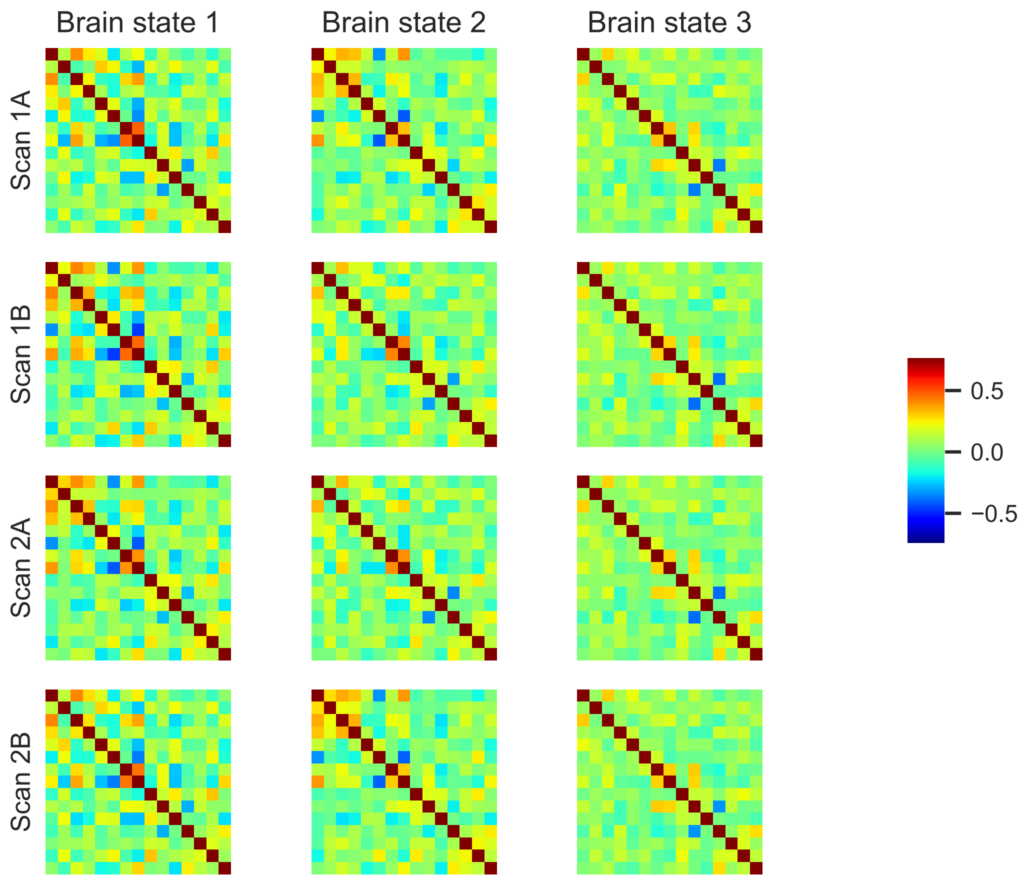

Fig. S9. Human Connectome Project (HCP) extracted brain states from dynamic conditional correlation, trained in joint ('-J') fashion (DCC-J) estimates.

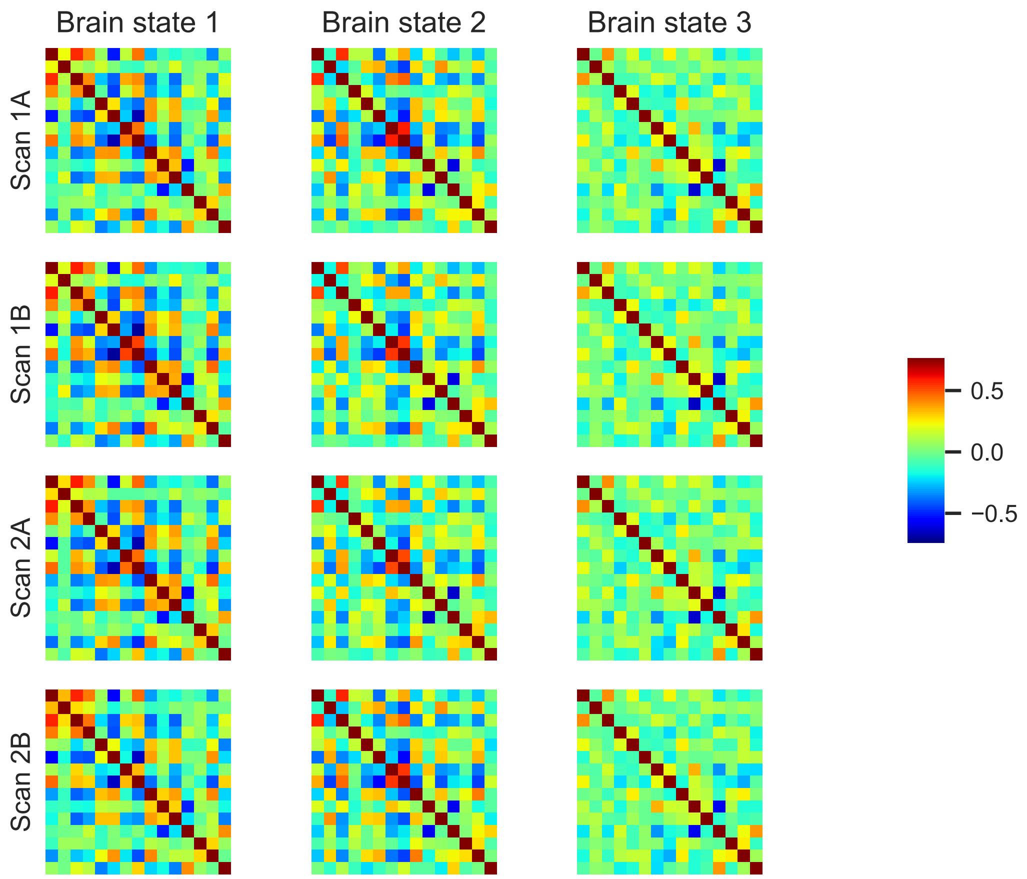

Fig. S10. Human Connectome Project (HCP) extracted brain states from cross-validated sliding windows (SW-CV) estimates.

S.6 Rockland data visualization

Here, we provide further visualizations of the Rockland data (Nooner et al., 2012). Fig. S11A shows how the activity of the visual areas was more correlated with the external visual stimulus task conditions, as witnessed by the BOLD observations from the visual areas tracking the external stimulus time series. Fig. S11B shows that the overall connectivity strength between visual areas is strong, and stronger than the connectivity between visual areas and the mPFC and M1 areas.

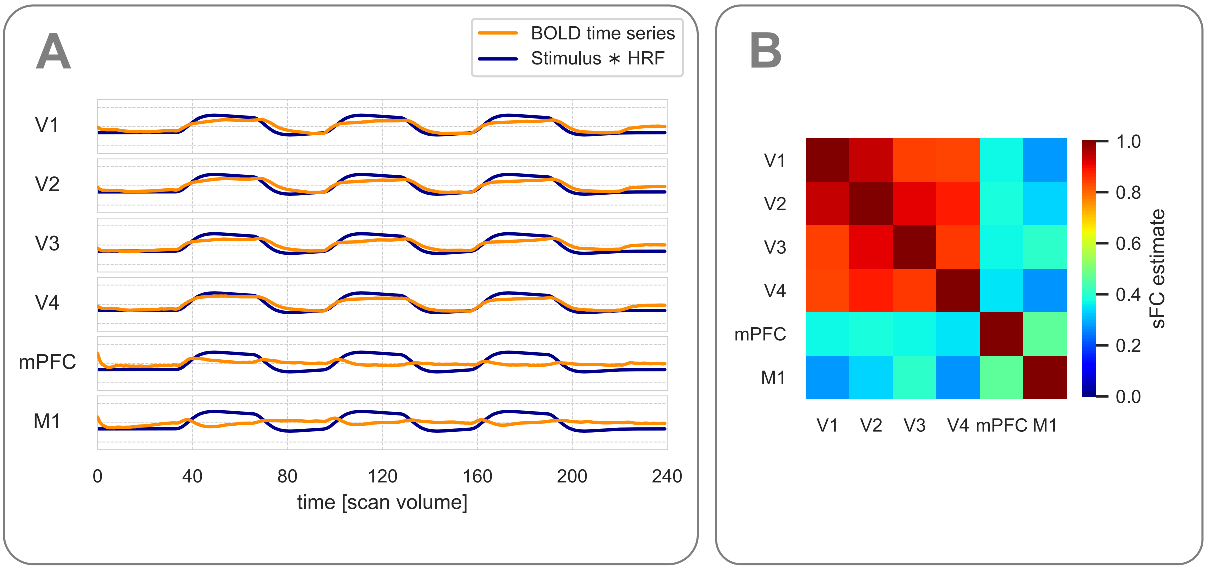

Fig. S11. (**A**) Rockland tb-fMRI data normalized BOLD time series, averaged over 286 participants. The external visual stimulus convolved with a hemodynamic response function (HRF) is shown for reference. The correspondence to the external stimulus decreased as we moved up and away from the visual processing hierarchy. (**B**) sFC (correlation) estimate averaged over all participants. The connectivity between V1 and other visual areas was stronger than with non-visual areas. Abbreviations: V1-V4; visual cortex regions, mPFC; medial prefrontal cortex, M1; primary motor cortex.

S.7 Relationship between window lengths and kernel lengthscales

In Section S.1, we validated that the cross-validation of SW window lengths leads to improved covariance estimation. Here, we discuss the relationship between the learned window lengths and WP kernel lengthscales (Eq. 2.14). As hypothesized in the introduction of the WP model, these two model parameters (l and $\hat{\omega}$) may capture similar aspects of covariance structures.

Across the simulation benchmarks, a longer window length is learned for the more static covariance structures (e.g., **null** and **constant**) (Fig. S12A). Shorter windows are learned for the covariance structures that change faster over time (e.g., the **periodic** covariance structures). Similarly, with the learned kernel lengthscales of the WP, the more static the covariance structure is, the larger the learned lengthscales (Fig. S12B). The faster the covariance structure changes over time, the smaller the lengthscales become. Note that the large values learned for the state transition covariance structure indicate that the models were not able to learn this structure. These findings suggest that these two hyperparameters capture similar dynamic aspects of covariance structures. However, the optimal window lengths were less distinct than the learned kernel lengthscales. For example, given the kernel lengthscales, it is possible to perfectly distinguish between the slow and fast periodic covariance structures, whereas this is not possible for window lengths.

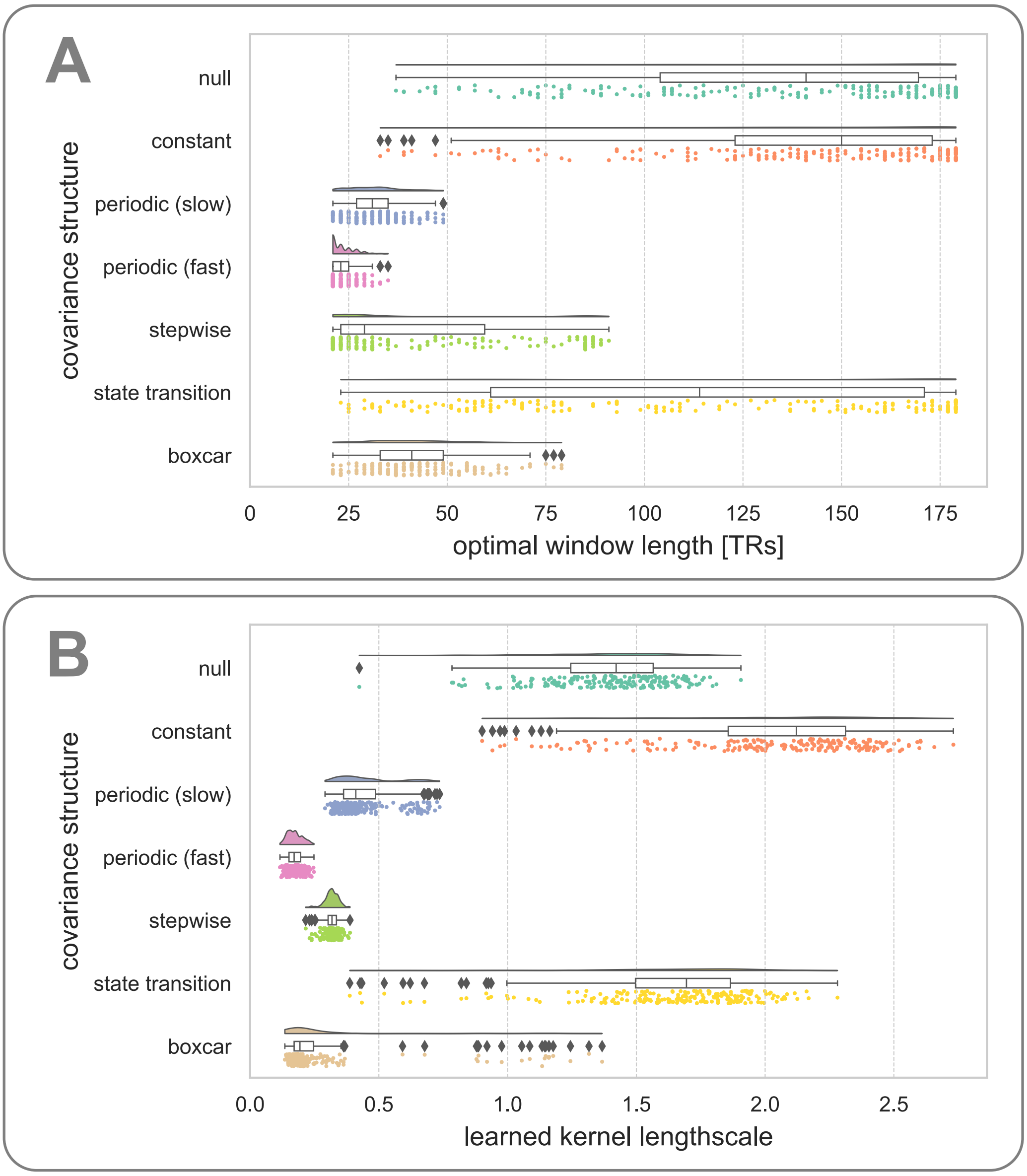

Fig. S12. Demonstration of adaptive hyperparameters in WP and SW-CV models. (**A**) Raincloud plot of simulation benchmarks cross-validated window lengths learned from bivariate noiseless data for $N = 400$. A faster change in covariance structure results in shorter learned window lengths. (**B**) Raincloud plot of simulation benchmarks WP kernel lengthscales learned from bivariate noiseless data for $N = 400$. Faster changing covariance structures result in shorter learned kernel lengthscales. Shown are the distribution of model hyperparameters (i.e., cross-validated window length and WP kernel lengthscale), a boxplot showing the median, quartiles, and outliers, and a scatterplot underneath each boxplot representing one of $T = 200$ trials. Abbreviations: WP; Wishart process, SW-CV; cross-validated sliding windows.

Next, we compared the learned WP kernel lengthscales with the estimated optimal window lengths in the HCP benchmark (Fig. S13A). Despite prior evidence that these two hyperparameters capture similar aspects of the data, we did not find any significant relationship between them across all the scans. The synthetic covariance structures used in the simulation benchmarks may simply have been more distinct, whereas in the HCP, the dynamics between participants may not have differed as much. Additionally, these hyperparameters may not be as crucial to the estimates, which could be driven by actual observations. We performed the same procedure for

the tb-fMRI Rockland benchmark (Fig. S13B). Again, we did not find a positive relationship between these two parameters. While our intuition on these two hyperparameters held in the edge cases of the synthetic covariance structures, we conclude that caution is advised when interpreting the learned model parameters in real data.

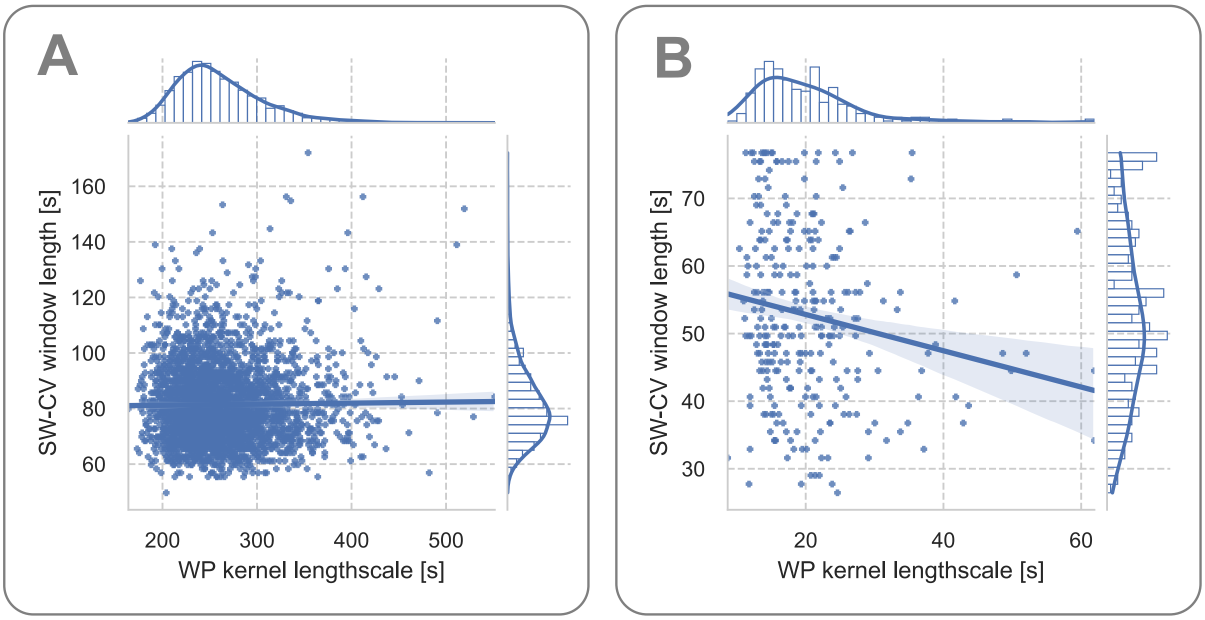

Fig. S13. Relationship between the learned window length and WP kernel lengthscales. (**A**) Human Connectome Project rs-fMRI data relationship between learned WP kernel lengthscales (scaled to time series length) and SW-CV optimal window length. Each dot represents one of the four scans (1A, 1B, 2A, 2B) of a single participant. The full time series was 864 seconds long. (**B**) Rockland tb-fMRI benchmark relationship between learned WP kernel lengthscales (scaled to time series length) and SW-CV optimal window length. Each dot represents one of the 286 Rockland participants. The full time series was 154.8 seconds long.

REFERENCES

Damoiseaux, J. S. (2017). Effects of aging on functional and structural brain connectivity. *NeuroImage*, *160*, 32–40. https://doi.org/10.1016/j.neuroimage.2017.01.077

Dhamala, E., Jamison, K. W., Jaywant, A., Dennis, S., & Kuceyeski, A. (2021). Distinct functional and structural connections predict crystallised and fluid cognition in healthy adults. *Human Brain Mapping*, *42*(10), 3102–3118. https://doi.org/10.1002/hbm.25420

Greene, A. S., Gao, S., Scheinost, D., & Constable, R. T. (2018). Task-induced brain state manipulation improves prediction of individual traits. *Nature Communications*, *9*(1). https://doi.org/10.1038/s41467-018-04920-3

Heaton, R. K., Akshoomoff, N., Tulsky, D., Mungas, D., Weintraub, S., Dikmen, S., Beaumont, J., Casaletto, K. B., Conway, K., Slotkin, J., & Gershon, R. (2014). Reliability and validity of composite scores from the NIH toolbox cognition battery in adults. *Journal of the International Neuropsychological Society*, *20*(6), 588–598. https://doi.org/10.1017/S1355617714000241

Li, J., Kong, R., Liégeois, R., Orban, C., Tan, Y., Sun, N., Holmes, A. J., Sabuncu, M. R., Ge, T., & Yeo, B. T. T. (2019). Global signal regression strengthens

association between resting-state functional connectivity and behavior. *NeuroImage*, *196*, 126–141. https://doi.org/10.1016/j.neuroimage.2019.04.016

Nooner, K. B., Colcombe, S. J., Tobe, R. H., Mennes, M., Benedict, M. M., Moreno, A. L., Panek, L. J., Brown, S., Zavitz, S. T., Li, Q., Sikka, S., Gutman, D., Bangaru, S., Schlachter, R. T., Kamiel, S. M., Anwar, A. R., Hinz, C. M., Kaplan, M. S., Rachlin, A. B., … Milham, M. P. (2012). The NKI-Rockland sample: A model for accelerating the pace of discovery science in psychiatry. *Frontiers in Neuroscience*, *6*. https://doi.org/10.3389/fnins.2012.00152

Pedregosa, F., Varoquaux, G., Gramfort, A., Michel, V., Thirion, B., Grisel, O., Blondel, M., Prettenhofer, P., Weiss, R., Dubourg, V., Vanderplas, J., Passos, A., Cournapeau, D., Brucher, M., Perrot, M., & Duchesnay, E. (2011). Scikit-learn: Machine Learning in Python. *Journal of Machine Learning Research*, *12*, 2825–2830. https://doi.org/10.1289/EHP4713

Smith, S. M., Nichols, T. E., Vidaurre, D., Winkler, A. M., Behrens, T. E. J., Glasser, M. F., Ugurbil, K., Barch, D. M., Van Essen, D. C., & Miller, K. L. (2015). A positive-negative mode of population covariation links brain connectivity, demographics and behavior. *Nature Neuroscience*, *18*(11), 1565–1567. https://doi.org/10.1038/nn.4125

Van Essen, D. C., Smith, S. M., Barch, D. M., Behrens, T. E. J., Yacoub, E., & Ugurbil, K. (2013). The WU-Minn Human Connectome Project: An overview. *NeuroImage*, *80*, 62–79. https://doi.org/10.1016/j.neuroimage.2013.05.041

Van Essen, D. C., Ugurbil, K., Auerbach, E., Barch, D., Behrens, T. E. J., Bucholz, R., Chang, A., Chen, L., Corbetta, M., Curtiss, S. W., Della Penna, S., Feinberg, D., Glasser, M. F., Harel, N., Heath, A. C., Larson-Prior, L., Marcus, D., Michalareas, G., Moeller, S., … Yacoub, E. (2012). The Human Connectome Project: A data acquisition perspective. *NeuroImage*, *62*, 2222–2231. https://doi.org/10.1016/j.neuroimage.2012.02.018

Weis, S., Patil, K. R., Hoffstaedter, F., Nostro, A., Yeo, B. T. T., & Eickhoff, S. B. (2020). Sex classification by resting state brain connectivity. *Cerebral Cortex*, *30*, 824–835. https://doi.org/10.1093/cercor/bhz129

Zamani Esfahlani, F., Faskowitz, J., Slack, J., Mišić, B., & Betzel, R. F. (2022). Local structure-function relationships in human brain networks across the lifespan. *Nature Communications*, *13*(1). https://doi.org/10.1038/s41467-022-29770-y
